# Decision Support Intervention and Anticoagulation for Emergency Department Atrial Fibrillation: The O’CAFÉ Stepped-Wedge Cluster Randomized Clinical Trial

**DOI:** 10.1001/jamanetworkopen.2024.43097

**Published:** 2024-11-06

**Authors:** David R. Vinson, E. Margaret Warton, Edward J. Durant, Dustin G. Mark, Dustin W. Ballard, Erik R. Hofmann, Dana R. Sax, Mamata V. Kene, James S. Lin, Luke S. Poth, Meena S. Ghiya, Anuradha Ganapathy, Patrick M. Whiteley, Sean C. Bouvet, Adina S. Rauchwerger, Jennifer Y. Zhang, Judy Shan, Daniel D. DiLena, Bory Kea, Ashok P. Pai, Jodi B. Loyles, Matthew D. Solomon, Alan S. Go, Mary E. Reed

**Affiliations:** 1 The Permanente Medical Group, Oakland, California; 2 Division of Research, Kaiser Permanente Northern California, Oakland; 3 Department of Emergency Medicine, Kaiser Permanente Roseville Medical Center, Roseville, California; 4 Department of Emergency Medicine, Kaiser Permanente Modesto Medical Center, Modesto, California; 5 Department of Emergency Medicine, Kaiser Permanente Oakland Medical Center, Oakland, California; 6 Department of Emergency Medicine, Kaiser Permanente San Rafael Medical Center, San Rafael, California; 7 Department of Emergency Medicine, Kaiser Permanente South Sacramento Medical Center, Sacramento, California; 8 Department of Emergency Medicine, Kaiser Permanente San Leandro Medical Center, San Leandro, California; 9 Department of Emergency Medicine, Kaiser Permanente Santa Clara Medical Center, Santa Clara, California; 10 Department of Emergency Medicine, Kaiser Permanente South San Francisco Creek Medical Center, San Francisco, California; 11 Department of Emergency Medicine, Kaiser Permanente San Jose Medical Center, San Jose, California; 12 Department of Emergency Medicine, Kaiser Permanente San Francisco Medical Center, San Francisco, California; 13 Harvard T.H. Chan School of Public Health, Boston, Massachusetts; 14 School of Medicine, University of California, San Francisco; 15 Center for Policy and Emergency Medicine, Department of Emergency Medicine, Oregon Health & Sciences University, Portland; 16 Department of Hematology and Oncology, Oakland Medical Center, Oakland, California; 17 Kaiser Permanente Northern California Regional Pharmacy, Oakland; 18 Department of Cardiology, Oakland Medical Center, Oakland, California; 19 Departments of Epidemiology, Biostatistics, and Medicine, University of California, San Francisco

## Abstract

**Importance:**

Oral anticoagulation for adults with atrial fibrillation or atrial flutter (AFF) who are at elevated stroke risk reduces the incidence of ischemic stroke but remains underused. Efforts to increase anticoagulation initiation on emergency department (ED) discharge have yielded conflicting results.

**Objective:**

To evaluate the effectiveness of a multipronged intervention supporting anticoagulation initiation for eligible adult ED patients.

**Design, Setting, and Participants:**

The Clinical Decision Support to Optimize Care of Patients With Atrial Fibrillation or Flutter in the Emergency Department (O’CAFÉ) pragmatic, stepped-wedge cluster randomized clinical trial was conducted from July 1, 2021, through April 30, 2023, at 13 community medical centers (in 9 clusters) of an integrated health system in Northern California. The study included adult ED patients with primary AFF eligible for anticoagulation initiation when discharged home. Clusters were randomly assigned to staggered dates for 1-way crossover from the control phase (usual care) to the intervention phase.

**Intervention:**

Physician education, facility-specific audit and feedback, and access to decision support, which identified eligible patients and recommended shared decision-making, anticoagulation initiation (if suitable), and timely follow-up.

**Main Outcomes and Measures:**

The main outcome was a composite of anticoagulation on discharge or within 30 days. A primary intention-to-treat analysis (decision support access regardless of use) and a secondary per-protocol analysis (decision support use) were performed. Multivariable analyses adjusted for intervention and exposure months with random effects, accounting for clustering by facility and patient.

**Results:**

A total of 3388 eligible patients with atrial fibrillation were discharged home: 2185 (64.5%) were receiving pre-ED arrival anticoagulation and 1203 (35.5%) were eligible for anticoagulation. Among the 1203 patients with an initiation-eligible encounter, the median age was 74.0 (IQR, 68.0-82.0) years and approximately half (618 [51.4%]) were men. Among the 387 patients with an initiation-eligible control encounter, 244 (63.0%) received anticoagulation (190 [49.0%] at discharge and 54 [14.0%] within 30 days). Among the 816 patients with an initiation-eligible intervention encounter, 558 (68.4%) received anticoagulation (428 [52.5%] on discharge and 130 [15.9%] within 30 days). There was no statistically significant change in initiation of anticoagulation associated with the intervention (adjusted odds ratio, 1.33 [95% CI, 0.75-2.35]; *P* = .13). Decision support was used for 217 eligible case patients (26.6%) (per protocol) and was associated with a statistically significant change in anticoagulation initiation when compared with 599 patients for whom decision support was not used (164 [75.6%] vs 394 [65.8%]; *P* = .008).

**Conclusions and Relevance:**

In this trial, a multipronged intervention to facilitate thromboprophylaxis among eligible ED patients with AFF did not significantly increase anticoagulation initiation. Opportunities exist to further improve stroke prevention among ED patients with primary AFF.

**Trial Registration:**

ClinicalTrials.gov Identifier: NCT05009225

## Key Points

**Question:**

Does a multipronged physician-directed intervention with clinical decision support improve anticoagulation initiation for eligible adult emergency department patients with atrial fibrillation on discharge or within 30 days of discharge?

**Findings:**

In this stepped-wedge cluster randomized clinical trial conducted among 13 US community emergency departments (in 9 clusters), anticoagulation initiation improved slightly (from 63% to 68%) but this finding was not statistically significant. Decision support use was modest (27%).

**Meaning:**

Clinical decision support availability was not associated with higher rates of anticoagulation initiation in this trial; modest use of decision support may have contributed to its lack of effectiveness.

## Teaser

This stepped-wedge cluster randomized clinical trial evaluates a multipronged physician-directed intervention with clinical decision support of anticoagulation initiation for eligible adult emergency department patients.

## Introduction

Atrial fibrillation (AF) and atrial flutter are common cardiac arrhythmias that have a substantial negative effect on individuals and public health.^1,2^ Both contribute to impaired quality of life and increase risk of ischemic stroke, heart failure, disability, and death.^3^ Oral anticoagulants (OACs) can reduce ischemic stroke risk by two-thirds and mortality by 25%.^4,5,6^ Despite recommendations in clinical practice guidelines,^3,5,6,7^ an estimated 30% to 50% of eligible adults with AF or flutter (hereinafter, AFF) remain untreated with OACs, even following widespread availability of direct OACs that are highly effective for stroke prevention and safer than vitamin K antagonists (eg, warfarin).^8,9,10,11^

Patients with AFF often present to the emergency department (ED) for treatment. These encounters provide an opportunity to identify patients eligible for OAC initiation.^12^ Initiating OACs on ED discharge home is safe and associated with a mortality reduction.^13^ In a large study, ED patients with AF who received OACs on discharge were more likely to be taking anticoagulants 1 year later compared with those only referred for treatment.^14^ Like other clinicians, emergency medicine physicians often underprescribe OACs to eligible patients with AFF.^15,16,17,18,19^ Several ED studies have used clinical decision support systems (CDSSs) to increase OAC prescribing for eligible patients, with mixed results.^20,21,22,23,24^

We designed a low-intensity multipronged intervention to improve care of ED patients with primary AFF. The intervention promoted evidence-based best practices across the breadth of ED AFF clinical issues, providing recommendations on rate reduction, cardioversion, stroke prevention, and trigger identification and avoidance (eTable 1 in Supplement 1).^25^ The intervention included serial physician education, monthly facility-specific audit and feedback, and access to a web-based CDSS called RISTRA-AF (Risk Stratification-AF).

Emergency physicians hold a range of opinions on their role in long-term stroke prevention in AFF, particularly with regard to whether to inform patients of their stroke risk and either (1) defer prescribing to others or (2) initiate OACs.^12,26^ We designed the CDSS to accommodate this variation. The RISTRA-AF system prepopulated the CHA_2_DS_2_-VASc (congestive heart failure, hypertension, age 75 years or older, diabetes, stroke, vascular disease, age 65-74 years, and female sex) score^27^ to help physicians identify patients with AFF eligible for OACs. Physicians were encouraged to have a shared decision-making conversation with at-risk patients about stroke prevention and to facilitate timely outpatient follow-up.

To test the effectiveness of the intervention, we undertook this pragmatic, stepped-wedge cluster randomized clinical trial across 13 community EDs, examining OAC initiation among eligible patients on ED discharge and in the subsequent 30 days. We performed both (1) a primary intention-to-treat analysis of the intervention that included CDSS access (irrespective of use) and (2) a secondary per-protocol analysis of CDSS use vs nonuse during the intervention phase. We hypothesized that the intervention would increase the proportion of eligible adults with AFF who were prescribed OACs on ED discharge or within the following 30 days, and we also hypothesized that OAC initiation would increase with CDSS use.

## Methods

### Study Design and Setting

The Clinical Decision Support to Optimize Care of Patients With Atrial Fibrillation or Flutter in the Emergency Department (O’CAFÉ) pragmatic, stepped-wedge cluster randomized clinical trial of a multipronged intervention was undertaken over 22 months from July 1, 2021, through April 30, 2023.^25^ We used an open cohort design (eMethods 1 in Supplement 1). The O’CAFÉ trial had 2 primary aims: to increase OAC initiation in eligible ED patients with primary AFF discharged home (the aim described herein) and to reduce hospitalization of the larger ED primary AFF population (which will be reported separately).^25^ We selected a stepped-wedge approach because the educational program of a staggered rollout in our system was easier to implement than with a traditional parallel-group design.^25,28^ This design also expanded intervention exposure across all study EDs, which was desirable because the intervention was thought to be an improvement over usual care. In this pragmatic trial, we designed the intervention to be low intensity with minimal disruption of customary clinical patterns of care, closer to usual than ideal care.^29^ The trial protocol is presented in Supplement 2. The Kaiser Permanente (KP) Northern California Institutional Review Board approved the study with a waiver of informed patient consent because it was determined to be minimal risk. This study followed the Consolidated Standards of Reporting Trials (CONSORT) guideline extension for stepped-wedge cluster randomized clinical trials.^30^

The O’CAFÉ trial was undertaken in KP Northern California, an integrated health care delivery system that provides comprehensive care for more than 4.5 million members who are representative of the ethnoracial and socioeconomic diversity of surrounding local and state populations.^31,32^ Kaiser Permanente Northern California is a learning health care system with an applied research agenda and is supported by a comprehensive integrated electronic health record (EHR) (Epic; Epic Systems), which includes inpatient, outpatient, emergency, pharmacy, laboratory, and imaging data.^33,34^

Emergency departments were staffed by emergency medicine residency-trained, board-certified (or board-eligible) physicians. During the study period, treating physicians had access to a standard discharge order set for AFF OACs, which recommended dabigatran, on the system’s pharmacy formulary, as the first-line OAC if not contraindicated. Care of patients taking OACs was managed by a regional pharmacy-led, telephone-based anticoagulation service using structured protocols.^35^

**Figure 1.**
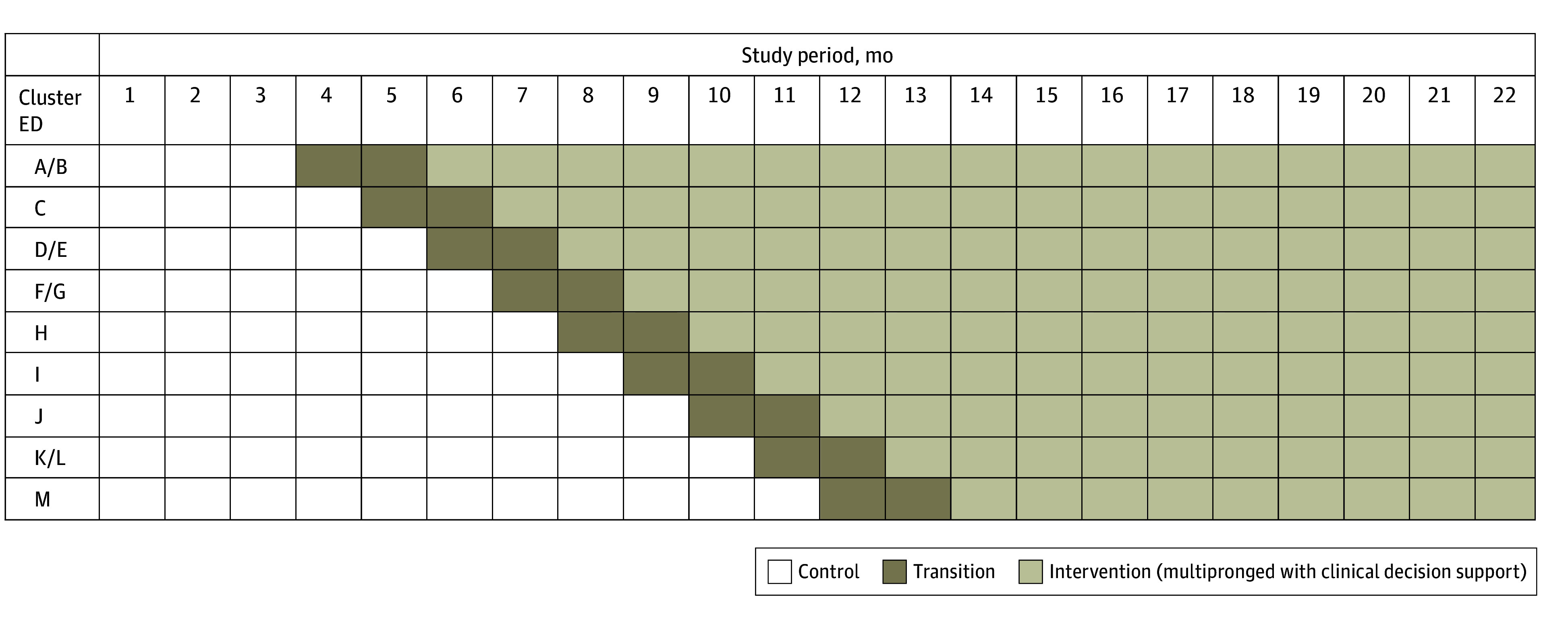
Time Course Over Which 9 Clusters Crossed Over From the Control Phase to the Intervention Phase Clusters were composed of 13 emergency departments (EDs), labeled A to M. Data in the transition phase were not analyzed. Adapted with permission from Vinson et al.^25^ Created by David R. Vinson, who holds the copyright (CC-BY-4.0).

The criteria for ED trial selection were as follows: (1) having an on-site emergency physician study champion and (2) not having participated in the pilot study. Pairs of EDs served by 1 shared staff of emergency physicians were treated as 1 site, leaving 9 study site clusters. The principal investigator allocated clusters to 1 of 9 sequences using a computer-generated randomization sequence. After an initial study period of 3 months in which all clusters were in the control phase, the intervention was implemented in 1 cluster per step at 1-month intervals, starting in October 2021 (Figure 1). The first 2 months of implementation were a transition period. The staggered rollout occurred over 9 months, followed by 11 months during which all clusters were in the transition or intervention phase.

### Study Population

**Figure 2.**
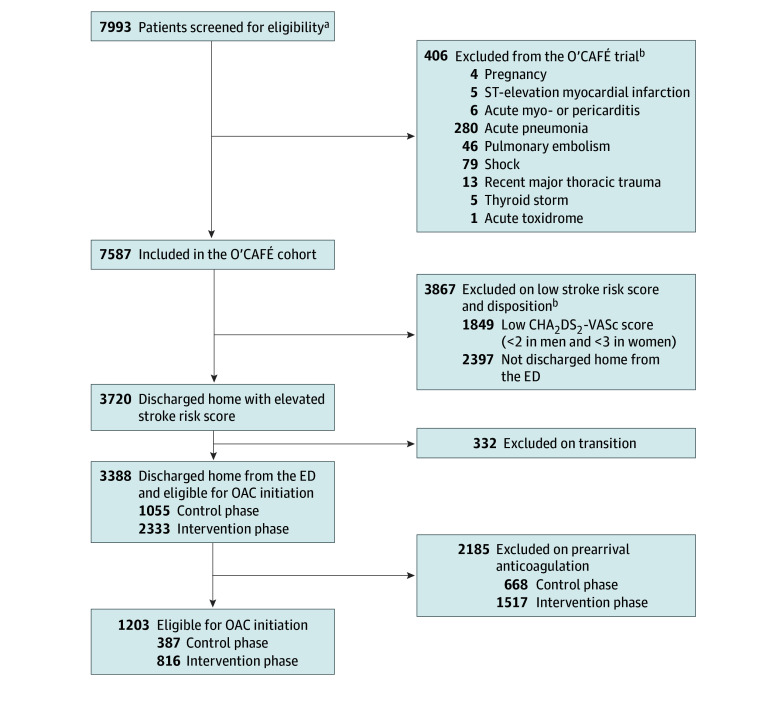
Cohort Assembly CHA_2_DS_2_-VASc indicates congestive heart failure, hypertension, age 75 years or older, diabetes, stroke, vascular disease, age 65 to 74 years, and female sex; ED, emergency department; OAC, oral anticoagulant; O’CAFÉ, Clinical Decision Support to Optimize Care of Patients With Atrial Fibrillation or Flutter in the Emergency Department. ^a^Adult member with primary diagnosis of atrial fibrillation or flutter (AFF) (also includes patients with a primary diagnosis of palpitations with a secondary diagnosis of AFF confirmed by 12-lead electrocardiography) in study ED from July 2021 through April 2023. ^b^Categories are nonexclusive, and encounters could have more than 1 exclusion condition.

The O’CAFÉ patients were adults (aged ≥18 years) receiving care for primary AFF in a participating ED during the study period (Figure 2), regardless of physician CDSS use. Patients with AFF were identified retrospectively using *International Statistical Classification of Diseases, Tenth Revision* diagnostic codes (I48.xx). Patients with AFF were excluded for any of the following concurrent ED diagnoses: pregnancy, ST-elevation myocardial infarction, acute myo- or pericarditis, acute pneumonia, pulmonary embolism, shock (eg, septic, hemorrhagic, or cardiogenic), recent major thoracic trauma (<48 hours), thyroid storm, or acute toxidrome (eg, sympathomimetic or anticholinergic). Patients with heart failure were not excluded to broaden the utility of the RISTRA-AF system. Criteria for inclusion in this stroke prevention analysis were an elevated CHA_2_DS_2_-VASc score (≥2 in men and ≥3 in women, as per 2019 US guidelines^6^), not currently or recently (<90 days) taking anticoagulants, and being discharged home from the ED (Figure 2).

### Multipronged Educational and Technology Intervention

Emergency department clinicians were blinded to the study until the month prior to or concurrent with gaining RISTRA-AF access, at which time they were educated about best practices for ED AFF management (eTable 1 in Supplement 1) and shown how to use the RISTRA-AF system.^25^ Throughout the intervention phase, standardized emails were distributed monthly to study EDs to acknowledge local CDSS users and highlight elements of recommended ED AFF management. Users who passed a brief training quiz on RISTRA-AF content received small gift cards for their first 3 enrollments, as with other studies.^36^ In June 2022, we began distributing to intervention site leads their department’s performance metrics on stroke prevention to share with their physicians. No outreach was undertaken among primary care clinicians.

**Figure 3.**
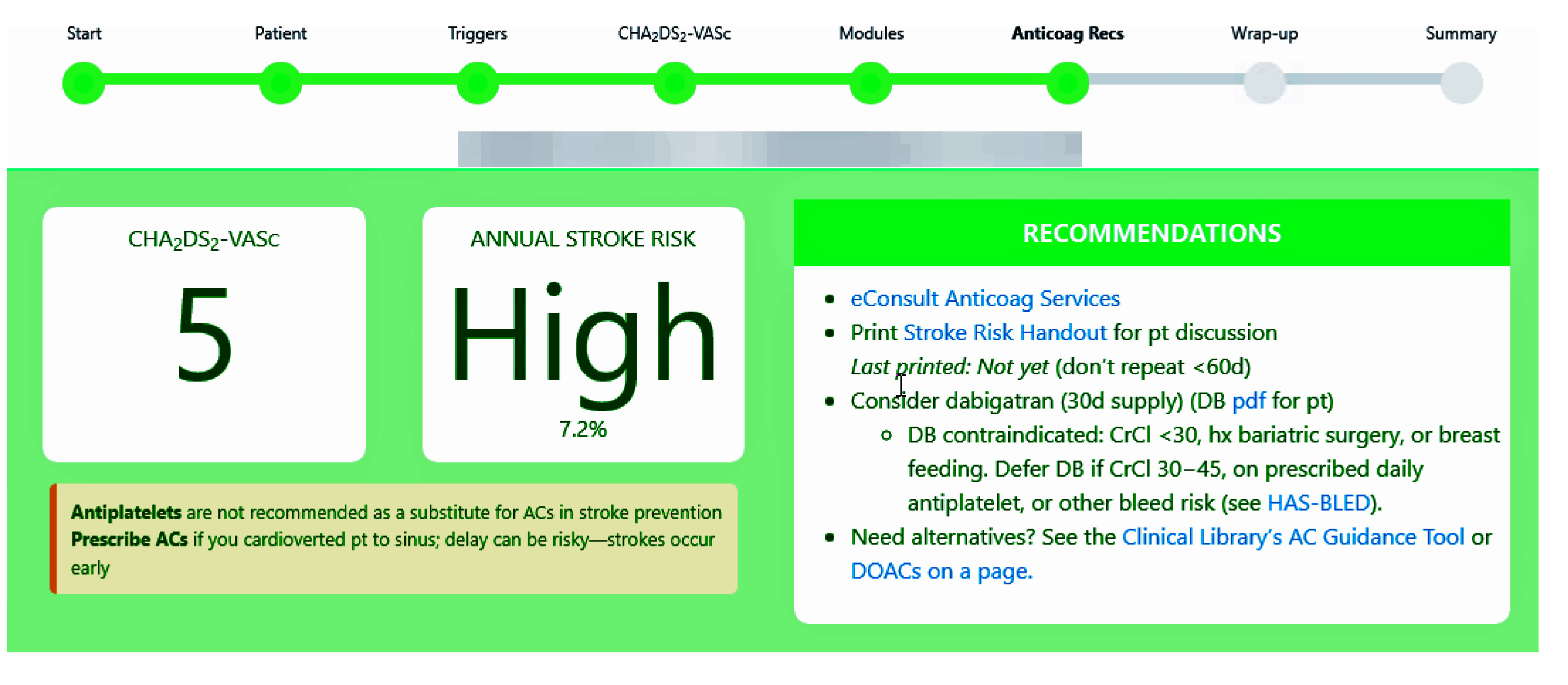
Anticoagulation Recommendations Screen From the Risk Stratification-AF (RISTRA-AF) Application for Patients With High Estimated Annual Risk for Ischemic Stroke Content of “hover to discover pop-ups” is as follows: eConsult Anticoag Services, “You can eConsult even when not prescribing if the pt wants more info on stroke prevention” (the eConsult was also a hyperlink to the consult order itself); consider dabigatran, “DB dose 150 mg BID; if age ≥80 y, use 110 mg.” AC indicates anticoagulant; Anticoag, anticoagulation; CrCl, creatinine clearance; DB, dabigatran; DOAC, direct oral anticoagulant; hx, history of; pt, patient. Adapted with permission from Vinson et al.^25^ Created by David R. Vinson and the KP CREST Network, who hold the copyright (CC-BY-4.0).

The RISTRA-AF system was accessible within the ED Navigator of the EHR but needed to be manually activated; no alert within the EHR reminded physicians of CDSS eligibility. Details of decision support leading up to anticoagulation recommendations are explained in eMethods 2, eFigure 1, and eFigure 2 in Supplement 1. The anticoagulation recommendation screen provided the patient’s CHA_2_DS_2_-VASc score, their estimated annual stroke risk, and several management options (Figure 3). The screen included an eConsult hyperlink to our health system’s pharmacy-led anticoagulation management service, which was indicated when prescribing OACs or when patients requested more information on the risks and benefits of anticoagulation. A patient-specific educational handout was available to be printed from the anticoagulation screen (eFigure 3 in Supplement 1). The clinician was also presented with age-based recommended doses of dabigatran, as well as common contraindications, and hyperlinks to a KP patient handout on dabigatran and to a KP table on alternative anticoagulants. The CDSS reminded physicians to consider bleed risk and to defer OACs if indicated (Figure 3). To help physicians estimate bleed risk, we provided a hyperlink to internal CDSS screens about the HAS-BLED (hypertension, abnormal kidney and/or liver function, stroke, bleeding history or predisposition, labile international normalized ratio, elderly [older age], and concomitant drug and/or alcohol use) scoring system, particularly its intent, its variables, and its score-based bleed risk estimates (eFigures 4 and 5 in Supplement 1).^37,38,39^

### Outcomes

Our primary outcome was a composite of OAC initiation among eligible patients with AFF, either on ED discharge or in the subsequent 30 days. Although the latter was not directly controlled by the treating emergency physician, the shared decision-making conversation with ED patients about stroke prevention may have facilitated post-ED discharge OAC initiation, as many patients wanted to continue the conversation with their primary care clinician. The study was designed to identify an anticipated 4.9% change in rates of OAC initiation with 80% power (detailed in eMethods 3 in Supplement 1).^25^

### Data Collection

We examined patient characteristics, including demographics, comorbidities, and CHA_2_DS_2_-VASc scores, as well as treatment variables, including use of the CDSS and OAC initiation within 30 days of ED discharge, using data extracted from EHRs and the RISTRA-AF system. Race and ethnicity was self-reported as Asian, Black or African American (hereinafter, Black), Hispanic or Latinx (hereinafter, Hispanic), White, or other race or ethnicity (including American Indian or Alaska Native or Native Hawaiian or Other Pacific Islander). Race and ethnicity data were collected to demonstrate that the diversity of the cohort reflects the diversity of the population of northern California. We randomly selected for manual review 40 case patients from the intervention phase in which OACs, although indicated, were not prescribed on ED discharge or within the subsequent 30 days. As in prior work, manual medical record review was conducted to examine unstructured documentation in the EHRs to identify treating ED physicians’ documentation of stroke risk and explanations for withholding OACs.^17^

### Statistical Analysis

We present categorical data as frequencies and proportions and continuous variables as medians with IQRs. We report binomial exact 95% CIs where appropriate. We examined within- and between-cluster correlation over time to elucidate possible correlation structures, including possible time decay in the correlation over time. We determined the intraclass correlation and churn rates over time and by cluster. We used descriptive statistics to examine outcome trends over time overall and by intervention status. We used marginal models (eMethods 4 in Supplement 1) to estimate the average treatment effect of the intervention on OAC initiation while allowing for clustering by patient and facility with appropriate correlation structures and adjustment for temporal trends and other time-related effects. We undertook a sensitivity analysis that included only each patient’s first eligible encounter. We performed both (1) a primary intention-to-treat analysis (decision support access regardless of use) and (2) a secondary per-protocol analysis (decision support use). We also compared 2 groups within the intervention phase: encounters without and with CDSS use, a preplanned analysis. Both groups received the same CDSS access intervention but differed in clinician CDSS use. The latter served as a per-protocol analysis to evaluate the effectiveness of CDSS use. *P* < .05 (2-tailed) was considered statistically significant. All analyses were conducted with SAS, version 9.4 (SAS Institute Inc).

## Results

A total of 3388 case patients meeting criteria for OACs were discharged from the ED during the study period: 2185 (64.5%) were receiving OACs before ED arrival and 1203 (35.5%) were eligible for OAC initiation. The 1203 initiation-eligible case patients (585 women [48.6%] and 618 men [51.4%]) had a median age of 74.0 (IQR, 68.0-82.0) years and a median CHA_2_DS_2_-VASc score of 4.0 (IQR, 3.0-5.0). Initiation-eligible case patients identified as Asian (163 [13.5%]), Black (71 [5.9%]), Hispanic (117 [9.7%]), White (803 [66.7%]), or other race or ethnicity (49 [4.1%]). A total of 387 patients (32.2%) were in the control phase and 816 (67.8%) were in the intervention phase. Patient characteristics were similar between the 2 study phases (Table).

**Table.** Emergency Department Patients With Primary Atrial Fibrillation or Atrial Flutter Eligible for Anticoagulation Initiation, Stratified by Study Phase^a^

Characteristic	Initiation-eligible patients (n = 1203)	Study phase	
		Control (n = 387)	Intervention (n = 816)
Age, y			
Mean (SD) [range]	74.6 (10.2) [27.0-101.0]	74.8 (10.2) [27.0-99.0]	74.5 (10.3) [38.0-101.0]
Median (IQR)	74.0 (68.0-82.0)	75.0 (68.0-82.0)	74.0 (68.0-81.0)
Category			
<65	163 (13.5)	54 (14.0)	109 (13.4)
65-74	447 (37.2)	138 (35.7)	309 (37.9)
≥75	593 (49.3)	195 (50.4)	398 (48.8)
Sex			
Female	585 (48.6)	179 (46.3)	406 (49.8)
Male	618 (51.4)	208 (53.7)	410 (50.2)
Race and ethnicity			
Asian	163 (13.5)	54 (14.0)	109 (13.4)
Black or African American	71 (5.9)	11 (2.8)	60 (7.4)
Hispanic or Latinx	117 (9.7)	38 (9.8)	79 (9.7)
White	803 (66.7)	270 (69.8)	533 (65.3)
Other^b^	49 (4.1)	14 (3.6)	35 (4.3)
Index atrial arrhythmia			
Atrial fibrillation	994 (82.6)	311 (80.4)	683 (83.7)
Atrial flutter or AFF	209 (17.4)	76 (19.6)	133 (16.3)
Comorbidity			
History of AFF	697 (57.9)	228 (58.9)	469 (57.5)
Hypertension	987 (82.0)	310 (80.1)	677 (83.0)
Vascular disease	847 (70.4)	270 (69.8)	577 (70.7)
Diabetes	377 (31.3)	116 (30.0)	261 (32.0)
Congestive heart failure	166 (13.8)	53 (13.7)	113 (13.8)
Ischemic stroke, transient ischemic attack, or thromboembolic disease	118 (9.8)	46 (11.9)	72 (8.8)
CHA_2_DS_2_-VASc score			
Mean (SD) [range]	4.0 (1.5) [2.0-9.0]	4.0 (1.6) [2.0-9.0]	4.0 (1.5) [2.0-9.0]
Median (IQR)	4.0 (3.0-5.0)	4.0 (3.0-5.0)	4.0 (3.0-5.0)
Category			
2-3	513 (42.6)	168 (43.4)	345 (42.3)
4-5	491 (40.8)	157 (40.6)	334 (40.9)
≥6	199 (16.5)	63 (16.3)	137 (16.8)

Abbreviations: AFF, atrial fibrillation or atrial flutter; CHA_2_DS_2_-VASc, congestive heart failure, hypertension, age 75 years or older, diabetes, stroke, vascular disease, age 65 to 74 years, and female sex.

^a^ Unless indicated otherwise, values are presented as No. (%) of patients.

^b^ Includes American Indian or Alaska Native and Native Hawaiian or Other Pacific Islander.

**Figure 4.**
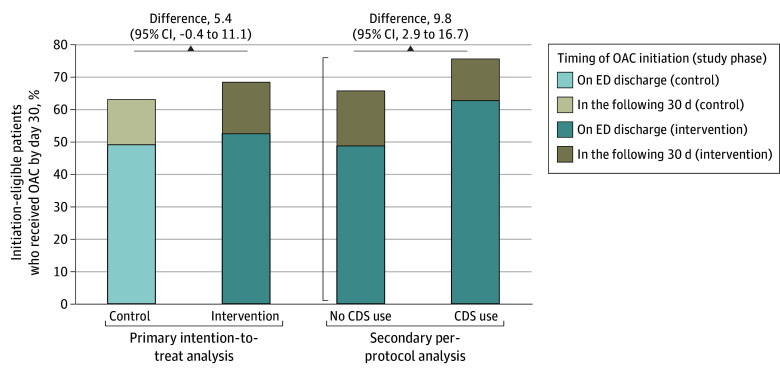
Anticoagulation Initiation on Emergency Department (ED) Discharge and in the Following 30 Days for Patients With Atrial Fibrillation or Flutter Meeting Criteria for Thromboprophylaxis During the Control and Intervention Phases CDS indicates clinical decision support; OAC, oral anticoagulant.

Among the 387 initiation-eligible control patients, 244 (63.0%) received OACs (190 [49.0%] on discharge and 54 [14.0%] within 30 days). Among the 816 initiation-eligible intervention case patients, 558 (68.4%) received OACs (428 [52.5%] on discharge and 130 [15.9%] within 30 days). The increase in OAC initiation from 63.0% (control phase) to 68.4% (intervention phase) was 5.4 percentage points (95% CI, −0.04 to 11.1; *P* = .07) (Figure 4). When adjusted, there was no statistically significant change in OAC initiation associated with the intervention (adjusted odds ratio, 1.33 [95% CI, 0.75 to 2.35]; *P* = .13). When patients receiving OACs before ED arrival were included, the increase in overall OAC use from 86.4% (control phase) to 88.9% (intervention phase) was 2.5 percentage points (95% CI, 0.07 to 4.92; *P* = .04). We report intraclass correlation and churn rates in eResults in Supplement 1. Sensitivity analysis including only first encounters resulted in minimal change to the odds ratio estimate and no change to the inference.

Among encounters in the intervention phase, patients receiving OACs had a lower prevalence of several comorbidities and a different distribution of CHA_2_DS_2_-VASc scores compared with untreated patients (eTable 2 in Supplement 1). During the intervention phase, physicians used the RISTRA-AF system with 217 eligible case patients (26.6%). Compared with 599 case patients for whom the CDSS was not used, those with physician CDSS use were dissimilar only with regard to history of prior AFF (eTable 3 in Supplement 1). In the per-protocol analysis, CDSS use was associated with a statistically significant increase in OAC initiation when compared with nonuse (164 [75.6%] vs 394 [65.8%]; *P* = .008) (Figure 4).

### Manual Medical Record Review of Sample Cases

We randomly selected 40 eligible case patients from the intervention phase for whom OACs were not initiated (on discharge or within 30 days) and conducted a manual medical record review. Twelve physicians’ clinical notes explained that after a shared decision-making discussion, their patients had declined anticoagulation. Eleven physicians noted elevated bleeding risks (eg, recent gastrointestinal bleed, pancytopenia, or upcoming major surgery). Nine physicians documented that their patients deferred the OAC decision until discussion with their primary care clinician. Six physicians did not mention stroke risk or OACs in their note. Finally, 2 physicians had undercalculated the CHA_2_DS_2_-VASc score (the method of score calculation was not documented).

## Discussion

In this pragmatic, stepped-wedge cluster randomized clinical trial conducted in a US community-based integrated health care system, a multipronged intervention including physician education, facility-specific audit and feedback, and CDSS access was not associated with a statistically significant change in OAC initiation on or within 30 days of ED discharge among patients with primary AFF. We address these results in light of our power calculations in eDiscussion 1 in Supplement 1.

We adopted a pragmatic trial design following recommendations from the PRECIS-2 (Pragmatic Explanatory Continuum Indicator Summary) tool.^29^ We sought to avoid major disruptions to usual care and to allow the intervention to be more easily adapted to less integrated care delivery settings. To illustrate, first, we addressed patients with typical AFF (including those with concomitant heart failure). Second, we kept changes to care delivery minimal. Third, stroke prevention recommendations were flexible and easy to implement (eg, making OAC initiation optional). Fourth, we did not embed an automated alert in the EHR. Fifth, CDSS activation was elective and physician driven. Finally, we used facility-specific rather than physician-specific audit and feedback, as we preferred a more sustainable approach and did not want physicians to feel their practice patterns were being scrutinized. These low-intensity characteristics supported the acceptability of the intervention in an ED environment whose baseline complexity was exacerbated by the concurrent COVID-19 pandemic. This streamlined implementation, however, may have reduced the likelihood of CDSS use and, therefore, the potential effectiveness of the intervention bundle on OAC initiation. These pragmatic accommodations may explain, in part, the relatively modest use of the CDSS.

To our knowledge, there has been only 1 other randomized clinical trial published to improve OAC initiation among eligible ED patients with AF discharged home. The Rapid Atrial Fibrillation/Flutter (RAFF-3) stepped-wedge cluster randomized clinical trial was conducted across 11 EDs in Canada, and it had a primary outcome of reducing ED length of stay.^23^ Improving OAC use was a secondary aim. In contrast to the O’CAFÉ trial, RAFF-3 excluded patients with permanent AF and those who had cardioverted spontaneously before initiation of ED treatment; more than 90% of enrolled patients had presumed recent-onset AF less than 48 hours in duration. Their preintervention OAC use was high: 61% of eligible patients received OACs before ED arrival and an additional 17% were prescribed OACs on discharge. The intervention included multimodal physician and nurse education of Canadian AF best practices^40,41^ (supported by a local champion with local action plans based on prior physician interviews that had identified barriers to implementation), a smartphone application, and facility-specific audit and feedback reports. The intervention failed to significantly increase OAC use on discharge. Initiation of OACs following discharge was not reported.

Several pre-to-post studies of ED OAC-focused CDSS interventions have been published, but methodological differences prevent ready comparison with our trial (eDiscussion 2 in Supplement 1).^20,21,22^ Forthcoming trials will be more comparable.^24^

On manual medical record review, we ascertained that most emergency physicians not prescribing OACs had a clinically acceptable reason for noninitiation (eg, the patient had a recent hemorrhage or upcoming surgery). After a shared decision-making discussion, some patients declined anticoagulation or wanted to continue the discussion with their primary care physician. A minority of patients, however, had their predicted stroke risk either unaddressed or even miscalculated, shortcomings in care that may have been reduced by more widespread CDSS use. Additional methods of enhancing ED physicians’ use of CDSS while maintaining a low-intensity approach need to be explored.^42^

### Limitations

This study had several limitations. Unlike some health systems, very high rates of baseline anticoagulation in the control phase of this study (64.5%) reduced the pool of patients with primary AFF eligible for OAC initiation. Thus, a larger sample may have been required, as other studies have found.^43^ We selected OAC initiation as our outcome because it focused on changing physician behavior for a highly effective preventive therapy. We did not study more patient-focused measures, such as filled prescriptions or longer-term ischemic stroke outcomes. Physicians who wanted assistance addressing stroke prevention may have been more likely to use the RISTRA-AF system, introducing selection bias. A Hawthorne effect was unavoidable: physicians during the intervention period may have increased their adherence to best practices knowing their behaviors were being studied. Stroke prevention is a critical component of comprehensive AFF care but may not be in the forefront of ED AFF management priorities while addressing more urgent issues of heart rate reduction and cardioversion. Fortunately, thromboprophylaxis in AFF is a multidisciplinary responsibility. Many different clinician touchpoints are necessary to optimize patient care. The trial’s integrated care delivery setting may limit generalizability of these findings, although the relatively low-intensity intervention involves components found in many quality improvement projects, making it more readily adaptable to a broad variety of settings.

## Conclusions

In this pragmatic, stepped-wedge cluster randomized clinical trial among 13 US community EDs, a multipronged educational and technical intervention did not significantly increase anticoagulation initiation. Opportunities exist to further improve stroke prevention among ED patients with primary AFF.

## Notes

Supplement 1. **eTable 1.** Management Recommendations Provided in the Clinical Decision Support Application RISTRA-AF to Improve Care of Emergency Department Patients with Primary Atrial Fibrillation or Flutter **eMethods 1.** Explanation of Open Cohort Design **eMethods 2.** Details of Decision Support Leading up to the Stroke Prevention Recommendations **eFigure 1.** Modules Screen of the RISTRA-AF Clinical Decision Support System **eFigure 2.** The CHA_2_DS_2_-VASc Screen of the RISTRA-AF Clinical Decision Support Application **eFigure 3.** Patient-Specific Handout Used in Shared Decision-Making on Stroke Prevention With At-Risk Emergency Department Patients With Atrial Fibrillation or Atrial Flutter **eFigure 4.** The Screen in RISTRA-AF That Explains the HAS-BLED Tool **eFigure 5.** The Screen in RISTRA-AF that Enumerates the Variables of the HAS-BLED Tool **eMethods 3.** Explanation of Power Calculation for the Larger O’CAFÉ Trial and the Stroke Prevention Aim **eMethods 4.** Explanation of Marginal Model **eResults.** Intraclass Correlation and Churn Rates **eTable 2.** Emergency Department Patients with Primary Atrial Fibrillation or Atrial Flutter Eligible for Anticoagulation Initiation on Intervention Condition, Stratified by Oral Anticoagulation Initiation on Discharge or Within 30 Days **eTable 3.** Emergency Department Patients With Primary Atrial Fibrillation or Atrial Flutter Eligible for Anticoagulation Initiation During Intervention Phase, Stratified by Clinical Decision Support Use **eDiscussion 1.** Results in the Context of Our Power Calculations **eDiscussion 2.** Pre-to-Post Studies of Anticoagulation-Focused Decision Support Interventions in the Emergency Department
eReferences

Supplement 2.
Trial Protocol

Supplement 3.
Data Sharing Statement

## References

[1] Rozen G, Hosseini SM, Kaadan MI, . Emergency department visits for atrial fibrillation in the United States: trends in admission rates and economic burden from 2007 to 2014. J Am Heart Assoc. 2018;7(15):e009024. doi:10.1161/JAHA.118.009024 30030215 PMC6201465

[2] Staerk L, Sherer JA, Ko D, Benjamin EJ, Helm RH. Atrial fibrillation: epidemiology, pathophysiology, and clinical outcomes. Circ Res. 2017;120(9):1501-1517. doi:10.1161/CIRCRESAHA.117.309732 28450367 PMC5500874

[3] Joglar JA, Chung MK, Armbruster AL, ; Writing Committee Members. 2023 ACC/AHA/ACCP/HRS guideline for the diagnosis and management of atrial fibrillation: a report of the American College of Cardiology/American Heart Association Joint Committee on Clinical Practice Guidelines. J Am Coll Cardiol. 2024;83(1):109-279. doi:10.1016/j.jacc.2023.08.017 38043043 PMC11104284

[4] Hart RG, Pearce LA, Aguilar MI. Meta-analysis: antithrombotic therapy to prevent stroke in patients who have nonvalvular atrial fibrillation. Ann Intern Med. 2007;146(12):857-867. doi:10.7326/0003-4819-146-12-200706190-00007 17577005

[5] January CT, Wann LS, Alpert JS, ; American College of Cardiology/American Heart Association Task Force on Practice Guidelines. 2014 AHA/ACC/HRS guideline for the management of patients with atrial fibrillation: a report of the American College of Cardiology/American Heart Association Task Force on Practice Guidelines and the Heart Rhythm Society. J Am Coll Cardiol. 2014;64(21):e1-e76. doi:10.1016/j.jacc.2014.03.022 24685669

[6] January CT, Wann LS, Calkins H, . 2019 AHA/ACC/HRS focused update of the 2014 AHA/ACC/HRS guideline for the management of patients with atrial fibrillation: a report of the American College of Cardiology/American Heart Association Task Force on Clinical Practice Guidelines and the Heart Rhythm Society. J Am Coll Cardiol. 2019;74(1):104-132. doi:10.1016/j.jacc.2019.01.011 30703431

[7] Hindricks G, Potpara T, Dagres N, ; ESC Scientific Document Group. 2020 ESC guidelines for the diagnosis and management of atrial fibrillation developed in collaboration with the European Association for Cardio-Thoracic Surgery (EACTS): the Task Force for the Diagnosis and Management of Atrial Fibrillation of the European Society of Cardiology (ESC) developed with the special contribution of the European Heart Rhythm Association (EHRA) of the ESC. Eur Heart J. 2021;42(5):373-498. doi:10.1093/eurheartj/ehaa612 32860505

[8] Søgaard M, Jensen M, Højen AA, . Net clinical benefit of oral anticoagulation among frail patients with atrial fibrillation: nationwide cohort study. Stroke. 2024;55(2):413-422. doi:10.1161/STROKEAHA.123.044407 38252753

[9] Munir MB, Hlavacek P, Keshishian A, . Oral anticoagulant underutilization among elderly patients with atrial fibrillation: insights from the United States Medicare database. J Interv Card Electrophysiol. 2023;66(3):771-782. 35804258 10.1007/s10840-022-01274-1 PMC10066118

[10] Navar AM, Kolkailah AA, Overton R, . Trends in oral anticoagulant use among 436 864 patients with atrial fibrillation in community practice, 2011 to 2020. J Am Heart Assoc. 2022;11(22):e026723. doi:10.1161/JAHA.122.026723 36346063 PMC9750070

[11] Al-Khatib SM, Pokorney SD, Al-Khalidi HR, . Underuse of oral anticoagulants in privately insured patients with atrial fibrillation: a population being targeted by the Implementation of a Randomized Controlled Trial to Improve Treatment With Oral Anticoagulants in Patients With Atrial Fibrillation (IMPACT-AFib). Am Heart J. 2020;229:110-117. doi:10.1016/j.ahj.2020.07.012 32949986 PMC8276627

[12] Vinson DR, Kea B, Coll-Vinent B, Barrett TW, Atzema CL. Enlisting emergency medicine clinicians to help reduce strokes in high-risk patients with atrial fibrillation and flutter. Clin Pharmacol Ther. 2018;104(4):613-614. doi:10.1002/cpt.1144 30006942 PMC6279237

[13] Coll-Vinent B, Martín A, Sánchez J, ; EMERG-AF Investigators. Benefits of emergency departments’ contribution to stroke prophylaxis in atrial fibrillation: the EMERG-AF study (Emergency Department Stroke Prophylaxis and Guidelines Implementation in Atrial Fibrillation). Stroke. 2017;48(5):1344-1352. doi:10.1161/STROKEAHA.116.014855 28389612 PMC5404399

[14] Atzema CL, Austin PC, Chong AS, Dorian P, Jackevicius CA. The long-term use of warfarin among atrial fibrillation patients discharged from an emergency department with a warfarin prescription. Ann Emerg Med. 2015;66(4):347-354.e2. doi:10.1016/j.annemergmed.2015.03.024 25964082

[15] Kea B, Waites BT, Lin A, . Practice gap in atrial fibrillation oral anticoagulation prescribing at emergency department home discharge. West J Emerg Med. 2020;21(4):924-934. doi:10.5811/westjem.2020.3.45135 32726266 PMC7390546

[16] Kea B, Alligood T, Robinson C, Livingston J, Sun BC. Stroke prophylaxis for atrial fibrillation? to prescribe or not to prescribe—a qualitative study on the decisionmaking process of emergency department providers. Ann Emerg Med. 2019;74(6):759-771. doi:10.1016/j.annemergmed.2019.03.026 31080035 PMC6842068

[17] Vinson DR, Warton EM, Mark DG, . Thromboprophylaxis for patients with high-risk atrial fibrillation and flutter discharged from the emergency department. West J Emerg Med. 2018;19(2):346-360. doi:10.5811/westjem.2017.9.35671 29560065 PMC5851510

[18] Yuguero O, Cabello I, Arranz M, . Emergency department capacity to initiate thromboprophylaxis in patients with atrial fibrillation and thrombotic risk after discharge: URGFAICS cohort analysis. Intern Emerg Med. 2022;17(3):873-881. doi:10.1007/s11739-021-02864-z 34677788

[19] Habeeb E, Papadopoulos T, Lewin AR, Knowles D. Assessment of anticoagulant initiation in patients with new-onset atrial fibrillation during emergency department visit-point-by-point response. Clin Appl Thromb Hemost. 2023;29:10760296231172493. doi:10.1177/10760296231172493 37138471 PMC10161192

[20] Rezazadeh S, Chew DS, Miller RJH, . Effects of a reminder to initiate oral anticoagulation in patients with atrial fibrillation/atrial flutter discharged from the emergency department: REMINDER study. CJEM. 2018;20(6):841-849. doi:10.1017/cem.2018.415 30295590

[21] Parkash R, Magee K, McMullen M, . The Canadian Community Utilization of Stroke Prevention Study in Atrial Fibrillation in the Emergency Department (C-CUSP ED). Ann Emerg Med. 2019;73(4):382-392. doi:10.1016/j.annemergmed.2018.09.001 30502307

[22] Barbic D, DeWitt C, Harris D, . Implementation of an emergency department atrial fibrillation and flutter pathway improves rates of appropriate anticoagulation, reduces length of stay and thirty-day revisit rates for congestive heart failure. CJEM. 2018;20(3):392-400. doi:10.1017/cem.2017.418 29117873

[23] Stiell IG, Archambault PM, Morris J, ; RAFF-3 Study Investigators. RAFF-3 trial: a stepped-wedge cluster randomised trial to improve care of acute atrial fibrillation and flutter in the emergency department. Can J Cardiol. 2021;37(10):1569-1577. doi:10.1016/j.cjca.2021.06.016 34217808

[24] Hopkins BJ, Nguyen T, Kinney E, . Optimizing stroke prophylaxis in the emergency department with an electronic clinical decision support tool: a preliminary analysis of a multi-stage multi-center stepped-wedge cluster randomized trial. Circulation. 2023;148(suppl 1):A18766. doi:10.1161/circ.148.suppl_1.18766

[25] Vinson DR, Rauchwerger AS, Karadi CA, ; Kaiser Permanente CREST Network. Clinical decision support to optimize care of patients with atrial fibrillation or flutter in the emergency department: protocol of a stepped-wedge cluster randomized pragmatic trial (O’CAFÉ trial). Trials. 2023;24(1):246. doi:10.1186/s13063-023-07230-2 37004068 PMC10064588

[26] Barrett TW, Marill KA. Anticoagulation for emergency department patients with atrial fibrillation: is our duty to inform or prescribe? Ann Emerg Med. 2013;62(6):566-568. doi:10.1016/j.annemergmed.2013.05.027 23810320

[27] Lip GY, Nieuwlaat R, Pisters R, Lane DA, Crijns HJ. Refining clinical risk stratification for predicting stroke and thromboembolism in atrial fibrillation using a novel risk factor-based approach: the Euro Heart Survey on Atrial Fibrillation. Chest. 2010;137(2):263-272. doi:10.1378/chest.09-1584 19762550

[28] Hemming K, Haines TP, Chilton PJ, Girling AJ, Lilford RJ. The stepped wedge cluster randomised trial: rationale, design, analysis, and reporting. BMJ. 2015;350:h391. doi:10.1136/bmj.h391 25662947

[29] Loudon K, Treweek S, Sullivan F, Donnan P, Thorpe KE, Zwarenstein M. The PRECIS-2 tool: designing trials that are fit for purpose. BMJ. 2015;350:h2147. doi:10.1136/bmj.h2147 25956159

[30] Hemming K, Taljaard M, McKenzie JE, . Reporting of stepped wedge cluster randomised trials: extension of the CONSORT 2010 statement with explanation and elaboration. BMJ. 2018;363:k1614. doi:10.1136/bmj.k1614 30413417 PMC6225589

[31] Gordon N, Lin T. The Kaiser Permanente Northern California Adult Member Health Survey. Perm J. 2016;20(4):15-225. doi:10.7812/TPP/15-225 27548806 PMC5101088

[32] Davis AC, Voelkel JL, Remmers CL, Adams JL, McGlynn EA. Comparing Kaiser Permanente members to the general population: implications for generalizability of research. Perm J. 2023;27(2):87-98. doi:10.7812/TPP/22.172 37170584 PMC10266863

[33] Bornstein S. An integrated EHR at Northern California Kaiser Permanente: pitfalls, challenges, and benefits experienced in transitioning. Appl Clin Inform. 2012;3(3):318-325. doi:10.4338/ACI-2012-03-RA-0006 23646079 PMC3613027

[34] Lieu TA, Platt R. Applied research and development in health care—time for a frameshift. N Engl J Med. 2017;376(8):710-713. doi:10.1056/NEJMp1611611 28225671

[35] An J, Niu F, Zheng C, . Warfarin management and outcomes in patients with nonvalvular atrial fibrillation within an integrated health care system. J Manag Care Spec Pharm. 2017;23(6):700-712. doi:10.18553/jmcp.2017.23.6.700 28530526 PMC10398296

[36] Vinson DR, Mark DG, Chettipally UK, ; eSPEED Investigators of the KP CREST Network. Increasing safe outpatient management of emergency department patients with pulmonary embolism: a controlled pragmatic trial. Ann Intern Med. 2018;169(12):855-865. doi:10.7326/M18-1206 30422263

[37] Pisters R, Lane DA, Nieuwlaat R, de Vos CB, Crijns HJ, Lip GY. A novel user-friendly score (HAS-BLED) to assess 1-year risk of major bleeding in patients with atrial fibrillation: the Euro Heart Survey. Chest. 2010;138(5):1093-1100. doi:10.1378/chest.10-0134 20299623

[38] Lip GY, Frison L, Halperin JL, Lane DA. Comparative validation of a novel risk score for predicting bleeding risk in anticoagulated patients with atrial fibrillation: the HAS-BLED (Hypertension, Abnormal Renal/Liver Function, Stroke, Bleeding History or Predisposition, Labile INR, Elderly, Drugs/Alcohol Concomitantly) score. J Am Coll Cardiol. 2011;57(2):173-180. doi:10.1016/j.jacc.2010.09.024 21111555

[39] Lip GY, Lane DA. Bleeding risk assessment in atrial fibrillation: observations on the use and misuse of bleeding risk scores. J Thromb Haemost. 2016;14(9):1711-1714. doi:10.1111/jth.13386 27296528

[40] Stiell IG, Scheuermeyer FX, Vadeboncoeur A, . CAEP acute atrial fibrillation/flutter best practices checklist. CJEM. 2018;20(3):334-342. doi:10.1017/cem.2018.26 34383280

[41] Stiell IG, de Wit K, Scheuermeyer FX, . 2021 CAEP Acute Atrial Fibrillation/Flutter Best Practices Checklist. CJEM. 2021;23(5):604-610. doi:10.1007/s43678-021-00167-y 34383280 PMC8423652

[42] Abell B, Naicker S, Rodwell D, . Identifying barriers and facilitators to successful implementation of computerized clinical decision support systems in hospitals: a NASSS framework-informed scoping review. Implement Sci. 2023;18(1):32. doi:10.1186/s13012-023-01287-y 37495997 PMC10373265

[43] Karlsson LO, Nilsson S, Bång M, Nilsson L, Charitakis E, Janzon M. A clinical decision support tool for improving adherence to guidelines on anticoagulant therapy in patients with atrial fibrillation at risk of stroke: a cluster-randomized trial in a Swedish primary care setting (the CDS-AF study). PLoS Med. 2018;15(3):e1002528. doi:10.1371/journal.pmed.1002528 29534063 PMC5849292

