# Supplementary material for: Decision Support Intervention and Anticoagulation for Emergency Department Atrial Fibrillation: The O’CAFÉ Stepped-Wedge Cluster Randomized Clinical Trial
Source: JAMA Netw Open. 2024 Nov 6;7(11):e2443097. doi: 10.1001/jamanetworkopen.2024.43097 (PMC11541643; doi:10.1001/jamanetworkopen.2024.43097)
Supplement: Supplement 1. — eTable 1. Management Recommendations Provided in the Clinical Decision Support Application RISTRA-AF to Improve Care of Emergency Department Patients with Primary Atrial Fibrillation or Flutter eMethods 1. Explanation of Open Cohort Design eMethods 2. Details of Decision Support Leading up to the Stroke Prevention Recommendations eFigure 1. Modules Screen of the RISTRA-AF Clinical Decision Support System eFigure 2. The CHA2DS2-VASc Screen of the RISTRA-AF Clinical Decision Support Application eFigure 3. Patient-Specific Handout Used in Shared Decision-Making on Stroke Prevention With At-Risk Emergency Department Patients With Atrial Fibrillation or Atrial Flutter eFigure 4. The Screen in RISTRA-AF That Explains the HAS-BLED Tool eFigure 5. The Screen in RISTRA-AF that Enumerates the Variables of the HAS-BLED Tool eMethods 3. Explanation of Power Calculation for the Larger O’CAFÉ Trial and the Stroke Prevention Aim eMethods 4. Explanation of Marginal Model eResults. Intraclass Correlation and Churn Rates eTable 2. Emergency Department Patients with Primary Atrial Fibrillation or Atrial Flutter Eligible for Anticoagulation Initiation on Intervention Condition, Stratified by Oral Anticoagulation Initiation on Discharge or Within 30 Days eTable 3. Emergency Department Patients With Primary Atrial Fibrillation or Atrial Flutter Eligible for Anticoagulation Initiation During Intervention Phase, Stratified by Clinical Decision Support Use eDiscussion 1. Results in the Context of Our Power Calculations eDiscussion 2. Pre-to-Post Studies of Anticoagulation-Focused Decision Support Interventions in the Emergency Department eReferences [file jamanetwopen-e2443097-s001.pdf]

Supplementary Online Content

Vinson DR, Warton EM, Durant EJ, et al. Decision support intervention and anticoagulation for emergency department atrial fibrillation: the O'CAFÉ stepped-wedge cluster randomized clinical trial. *JAMA Netw Open*. 2024;7(11):e2443097. doi:10.1001/jamanetworkopen.2024.43097

eTable 1. Management Recommendations Provided in the Clinical Decision Support Application RISTRA-AF to Improve Care of Emergency Department Patients with Primary Atrial Fibrillation or Flutter

eMethods 1. Explanation of Open Cohort Design

eMethods 2. Details of Decision Support Leading up to the Stroke Prevention Recommendations

eFigure 1. Modules Screen of the RISTRA-AF Clinical Decision Support System

eFigure 2. The CHA_2DS_2-VASc Screen of the RISTRA-AF Clinical Decision Support Application

eFigure 3. Patient-Specific Handout Used in Shared Decision-Making on Stroke Prevention With At-Risk Emergency Department Patients With Atrial Fibrillation or Atrial Flutter

eFigure 4. The Screen in RISTRA-AF That Explains the HAS-BLED Tool

eFigure 5. The Screen in RISTRA-AF That Enumerates the Variables of the HAS-BLED Tool

eMethods 3. Explanation of Power Calculation for the Larger O'CAFÉ Trial and the Stroke Prevention Aim

eMethods 4. Explanation of Marginal Model

eResults. Intraclass Correlation and Churn Rates

eTable 2. Emergency Department Patients with Primary Atrial Fibrillation or Atrial Flutter Eligible for Anticoagulation Initiation on Intervention Condition, Stratified by Oral Anticoagulation Initiation on Discharge or Within 30 Days

eTable 3. Emergency Department Patients With Primary Atrial Fibrillation or Atrial Flutter Eligible for Anticoagulation Initiation During Intervention Phase, Stratified by Clinical Decision Support Use

eDiscussion 1. Results in the Context of Our Power Calculations

eDiscussion 2. Pre-to-Post Studies of Anticoagulation-Focused Decision Support Interventions in the Emergency Department

eReferences

© 2024 Vinson DR et al. *JAMA Network Open.*

This supplementary material has been provided by the authors to give readers additional information about their work.

eTable 1. Management Recommendations Provided in the Clinical Decision Support Application RISTRA-AF to Improve Care of Emergency Department Patients with Primary Atrial Fibrillation or Flutter

Major Recommendations in Electronic Clinical Decision Support Application	Rationale for Recommendation
**1. Sustained rate reduction**	
Administer long-acting rate-reducing medications early in the ED encounter, either in addition to or in lieu of standard intravenous bolus medications	Medications with sustained effect on rapid ventricular response have been central to multifaceted ED interventions associated with reduced hospitalization.
**2. Effective cardioversion**	
**2A. Electrical**	
Start with maximal joules and consider manual pressure augmentation, especially for obese patients	These measures improve first-shock success and may reduce sedation duration and risk.
**2B. Pharmacologic**	
Consider efficiency in addition to effectiveness, safety, and ease of administration when selecting medications	For example, medications with a shorter time to effect, e.g., intravenous procainamide (median 30-40 min), facilitate ED operational efficiencies, unlike intravenous amiodarone, which does not distinguish itself from placebo for 6-8 hours.
**3. Stroke prevention**	
A. Identify patients at risk using auto-populating validated scoring system	Stroke risk stratification is the essential preparatory step for any subsequent stroke prevention action.
B. Print risk-specific handout for eligible patients and review with patient and family at bedside	The handout helps initiate a shared decision-making conversation on stroke prevention that can continue with outpatient physicians following discharge to home.
C1. Initiate outpatient anticoagulation at the time of ED discharge to home	Oral anticoagulation with DOACs or warfarin significantly reduces ischemic stroke and death in patients with AFF. Prescription on ED discharge can be associated with higher long-term use than when prescribing is left to post-discharge outpatient care.
C2. Or electronically consult the Anticoagulation Management Service to request they contact patients who want to learn more about stroke prevention before initiating anticoagulation	Following discharge to home, anticoagulation pharmacists can call interested patients to provide in-depth education on benefits and risks of anticoagulation for stroke prevention.
**4. Timely Follow-up**	
a. Encourage or request close follow-up (<7d) with outpatient physicians	Transferring care to outpatient physicians who can oversee longitudinal care of AFF and related conditions is key to long-term management

© 2024 Vinson DR et al. *JAMA Network Open.*

eTable 1. Management Recommendations Provided in the Clinical Decision Support Application RISTRA-AF to Improve Care of Emergency Department Patients with Primary Atrial Fibrillation or Flutter

Major Recommendations in Electronic Clinical Decision Support Application	Rationale for Recommendation
	success. Moreover, follow-up of these patients within a week of discharge has been associated with a reduction in the rate of death and hospitalization within 1 year.

AFF, atrial fibrillation or atrial flutter; DOAC, direct oral anticoagulant; ED, emergency department

Created by the authors of reference #1, who hold the copyright (CC-BY-4.0). Used with permission.

eMethods 1. Explanation of Open Cohort Design

The Stroke Prevention Aim of the O'CAFÉ trial was designed as an open-cohort study so that patients could technically have multiple eligible encounters during the study period. Patients who had only a single encounter would be counted as "churned", including patients who received the intervention, adhered to oral anticoagulation and thus were no longer eligible at subsequent AFF encounters. For this analysis we expected a high churn rate, with low numbers of patients with multiple eligible encounters. Among 1,203 eligible encounters in the study period, excluding washout periods, 1,149 patients had only 1 eligible ED encounter for a churn rate of 95.5%. The remaining 54 encounters included 47 patients with 2 eligible encounters and 7 patients with 3 or more encounters. We suspected these patients would be less likely to initiate recommended oral anticoagulation treatment, so we performed a sensitivity analysis including only the first encounter per patient. This resulted in minimal change to the odds ratio estimate and no change to the inference.

eMethods 2. Details of Decision Support Leading up to the Stroke Prevention Recommendations

Once activated, RISTRA-AF provided decision support on the leading clinical questions surrounding ED AFF management, e.g., rate control, cardioversion, and stroke prevention (**eTable 1; eFigure 1**). On the CHA_2DS_2-VASc screen (**eFigure 2**), demographic and clinical variables were pre-populated if available from the electronic health record. Clinician users reviewed the pre-populated entries and confirmed or edited as needed. The patient's CHA_2DS_2-VASc score was reported on this screen as well as the modules screen (**eFigure 1**). The stroke prevention module was not accessible if the physician had denoted earlier in RISTRA-AF that the patient was already on oral anticoagulation, if the patient had 1 of the 3 exclusions listed on the CHA_2DS_2-VASc screen (**eFigure 2**), or if the CHA_2DS_2-VASc score was below the thromboprophylaxis threshold (2 in men and 3 in women, as per U.S. guidelines).[2]

eFigure 1. Modules Screen of the RISTRA-AF Clinical Decision Support System

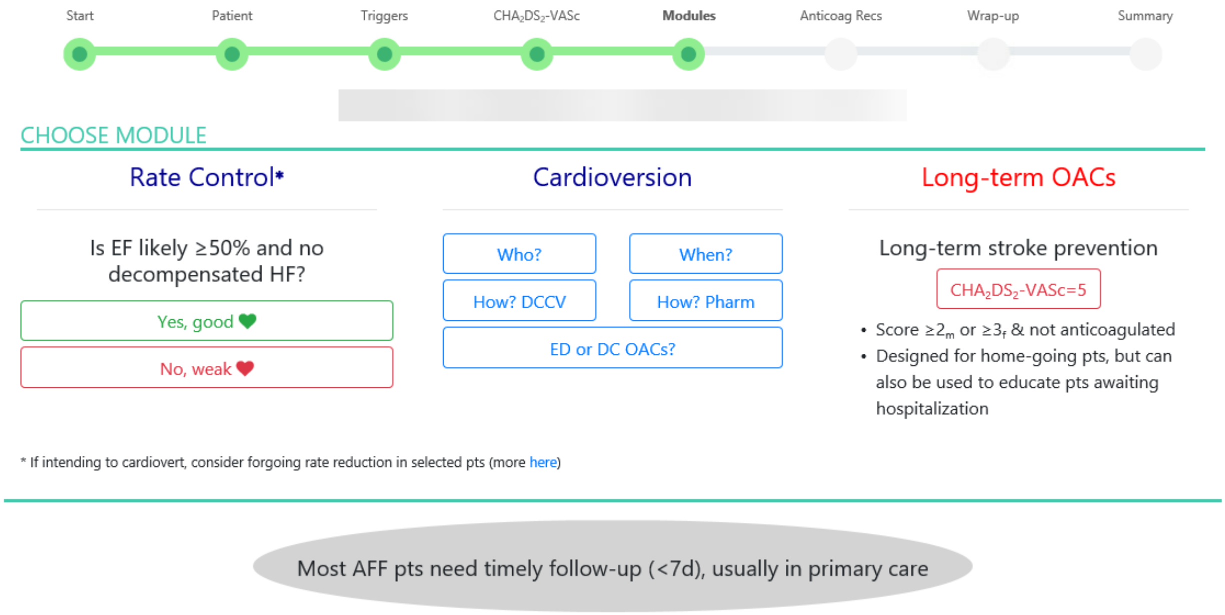

EF, ejection fraction; HF, heart failure; OAC, oral anticoagulation; pts, patients; ♥, heart.

Created by the authors of reference #1, who hold the copyright (CC-BY-4.0). Used with permission.

eFigure 2. The CHA_2DS_2-VASc Screen of the RISTRA-AF Clinical Decision Support Application

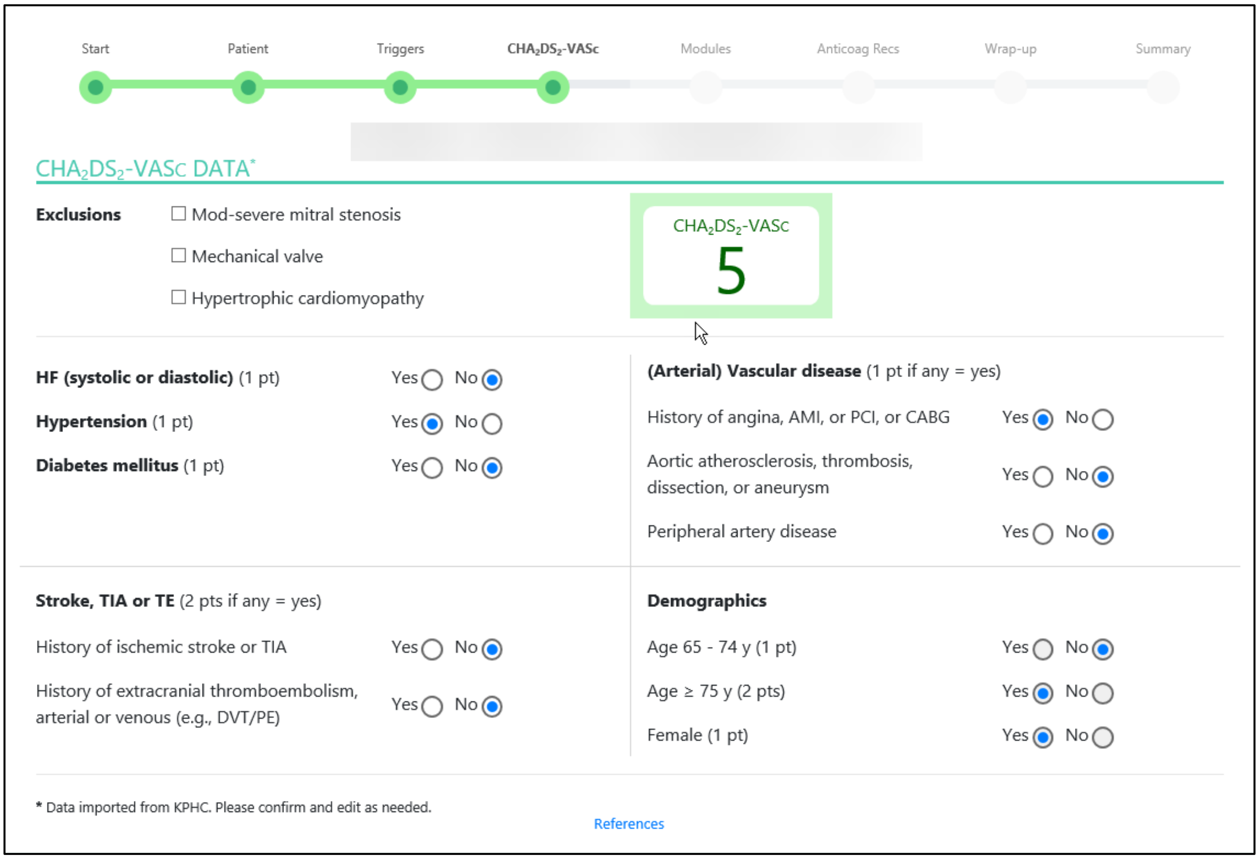

AMI, acute myocardial infarction; CABG, coronary artery bypass graft; DVT, deep vein thrombosis; HF, heart failure; PCI, percutaneous coronary intervention; PE, pulmonary embolism; pts, points; TE, thromboembolism; TIA, transient ischemic attack.

Created by the authors of reference #1, who hold the copyright (CC-BY-4.0). Used with permission.

eFigure 3. Patient-Specific Handout Used in Shared Decision-Making on Stroke Prevention With At-Risk Emergency Department Patients With Atrial Fibrillation or Atrial Flutter

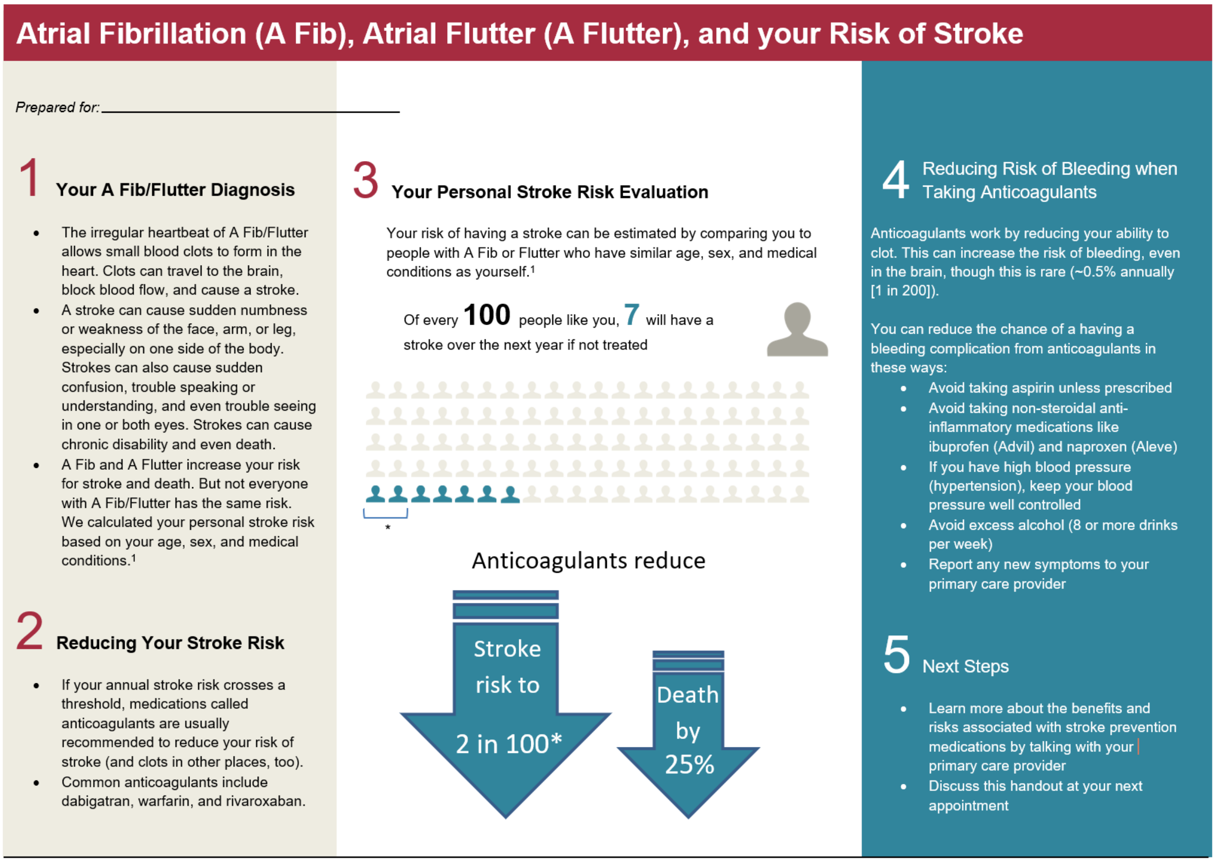

Atrial Fibrillation (A Fib), Atrial Flutter (A Flutter), and your Risk of Stroke

Prepared for: ______________________

1 Your A Fib/Flutter Diagnosis

- The irregular heartbeat of A Fib/Flutter allows small blood clots to form in the heart. Clots can travel to the brain, block blood flow, and cause a stroke.
- A stroke can cause sudden numbness or weakness of the face, arm, or leg, especially on one side of the body. Strokes can also cause sudden confusion, trouble speaking or understanding, and even trouble seeing in one or both eyes. Strokes can cause chronic disability and even death.
- A Fib and A Flutter increase your risk for stroke and death. But not everyone with A Fib/Flutter has the same risk. We calculated your personal stroke risk based on your age, sex, and medical conditions.[1]

2 Reducing Your Stroke Risk

- If your annual stroke risk crosses a threshold, medications called anticoagulants are usually recommended to reduce your risk of stroke (and clots in other places, too).
- Common anticoagulants include dabigatran, warfarin, and rivaroxaban.

3 Your Personal Stroke Risk Evaluation

Your risk of having a stroke can be estimated by comparing you to people with A Fib or Flutter who have similar age, sex, and medical conditions as yourself.[1]

Of every **100** people like you, **7** will have a stroke over the next year if not treated

*

Anticoagulants reduce

Stroke risk to 2 in 100*

Death by 25%

4 Reducing Risk of Bleeding when Taking Anticoagulants

Anticoagulants work by reducing your ability to clot. This can increase the risk of bleeding, even in the brain, though this is rare (~0.5% annually [1 in 200]).

You can reduce the chance of a having a bleeding complication from anticoagulants in these ways:

- Avoid taking aspirin unless prescribed
- Avoid taking non-steroidal anti-inflammatory medications like ibuprofen (Advil) and naproxen (Aleve)
- If you have high blood pressure (hypertension), keep your blood pressure well controlled
- Avoid excess alcohol (8 or more drinks per week)
- Report any new symptoms to your primary care provider

5 Next Steps

- Learn more about the benefits and risks associated with stroke prevention medications by talking with your primary care provider
- Discuss this handout at your next appointment

Created by the authors of reference #1, who hold the copyright (CC-BY-4.0). Used with permission.

eFigure 4. The Screen in RISTRA-AF That Explains the HAS-BLED Tool

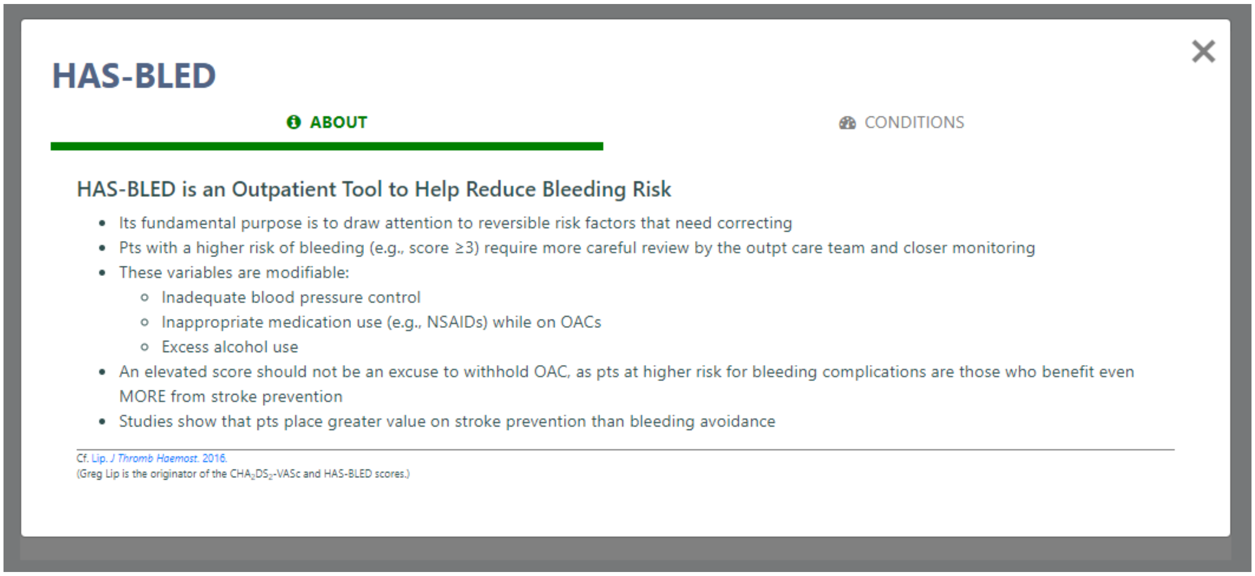

HAS-BLED

ABOUT | CONDITIONS

HAS-BLED is an Outpatient Tool to Help Reduce Bleeding Risk

- Its fundamental purpose is to draw attention to reversible risk factors that need correcting
- Pts with a higher risk of bleeding (e.g., score ≥3) require more careful review by the outpt care team and closer monitoring
- These variables are modifiable:
  - Inadequate blood pressure control
  - Inappropriate medication use (e.g., NSAIDs) while on OACs
  - Excess alcohol use
- An elevated score should not be an excuse to withhold OAC, as pts at higher risk for bleeding complications are those who benefit even MORE from stroke prevention
- Studies show that pts place greater value on stroke prevention than bleeding avoidance

Cf. Lip. *J Thromb Haemost*. 2016.
(Greg Lip is the originator of the CHA_2DS_2-VASc and HAS-BLED scores.)

NSAIDs, non-steroidal anti-inflammatory drugs; OACs, oral anticoagulants; outpt, outpatient; pts, patients.

Created by the authors of reference #1, who hold the copyright (CC-BY-4.0). Used with permission.

eFigure 5. The Screen in RISTRA-AF that Enumerates the Variables of the HAS-BLED Tool

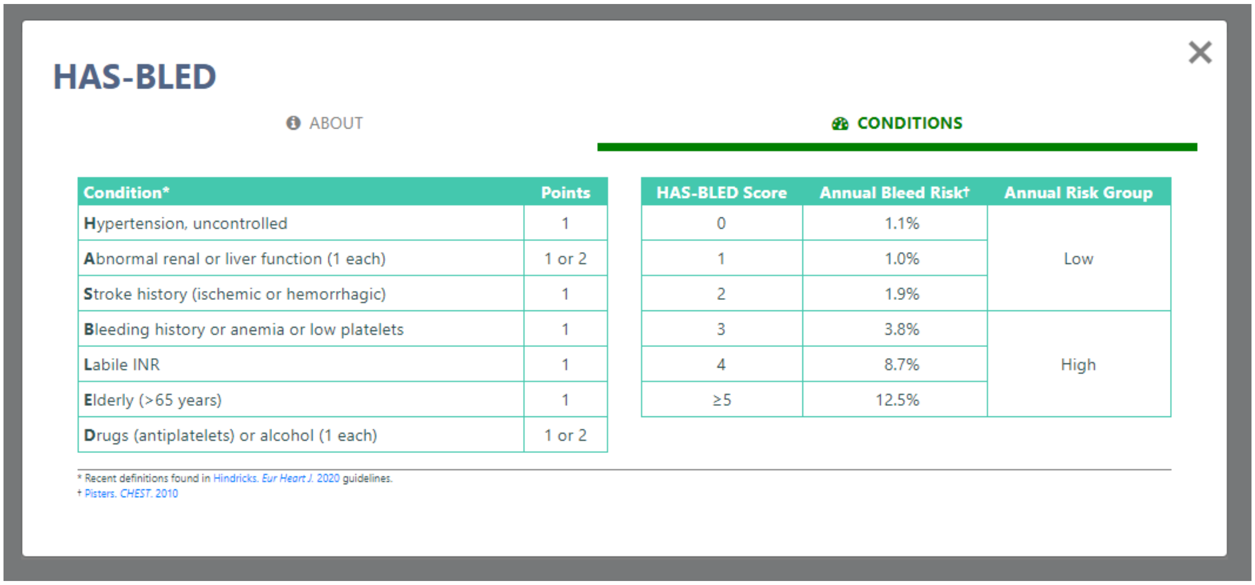

HAS-BLED

ABOUT | CONDITIONS

Condition*	Points
**H**ypertension, uncontrolled	1
**A**bnormal renal or liver function (1 each)	1 or 2
**S**troke history (ischemic or hemorrhagic)	1
**B**leeding history or anemia or low platelets	1
**L**abile INR	1
**E**lderly (>65 years)	1
**D**rugs (antiplatelets) or alcohol (1 each)	1 or 2

HAS-BLED Score	Annual Bleed Risk†	Annual Risk Group
0	1.1%	Low
1	1.0%	
2	1.9%	
3	3.8%	High
4	8.7%	
≥5	12.5%	

* Recent definitions found in Hindricks. *Eur Heart J*. 2020 guidelines.
† Pisters. *CHEST*. 2010

INR, international normalized ratio.

Created by the authors of reference #1, who hold the copyright (CC-BY-4.0). Used with permission.

eMethods 3. Explanation of Power Calculation for the Larger O'CAFÉ Trial and the Stroke Prevention Aim (excerpted from our methods paper published in *Trials*[1] with additional references)

We estimate that our stepped-wedge design (with 9 clusters and 10 steps) will include approximately 3,420 adult ED encounters with primary and 972 (30%) with isolated AF or atrial flutter during the 10-month roll-out period. Based on pilot data, we expect at least 567 patients in the usual care condition and 460 patients in the intervention condition with isolated AF or atrial flutter during the 10-month roll-out period. Using preliminary data at the pilot sites and the trial sites, baseline initial hospitalization rate was 26.6%. We estimate a minimally detectable 8% absolute difference in initial hospitalization rate (Aim 1) at a level of 90% power and a 2-sided test at the 2.5% significance level.

We estimated the minimum number of clusters needed to achieve 90% power based on pilot data using the National Institutes of Health Stepped-wedge Group Randomized Trial Calculator.[3-9] We present our most conservative estimates here. For the hospitalization outcome, we assume an average of 11 eligible encounters per cluster, intraclass correlation of 0.01, the cluster autocorrelation of 0.47, and the individual autocorrelation of 0.9 with a discrete-time decay, a churn-rate of 0.942 and adjustment for 1 cluster-level variable (annual ED census) with R^2 of 0.07. We would need only 3 clusters to see a decrease of 8% in hospitalization rates at 90% power, so we believe that we have adequate power in this analysis, given data from 9 clusters over the course of the trial.

Given that only 18% of ED encounters are eligible for stroke prevention action (discharged to home, current KP member, not currently or recently taking oral anticoagulants, and at high risk for stroke), the overall numbers of eligible encounters for the stroke-prevention related outcomes are much smaller. For the primary Aim 2 outcome (any prescription ordered for oral anticoagulation medications within 30 days of the index visit), power is still adequate in this study design to identify changes in rates of prescriptions ordered as small as 5% in the eligible subgroup. Based on pilot data and assuming an average of 7 eligible encounters per cluster, intraclass correlation of 0.006, the cluster autocorrelation of 0.356, and the individual autocorrelation coefficient of 0 with a discrete-time decay, a churn-rate of 0.984 with no adjustments for cluster-level variables, our 9-cluster design will allow us to identify a 4.9% change in rates of anticoagulant prescription with 80% power.

eMethods 4. Explanation of Marginal Model

We used the PROC GLIMMIX procedure in SAS to fit a marginal model that is similar to a generalized estimating equations model in that it accounts for correlation within clusters and generates robust standard errors.[10] We specified R-side random effects to model the correlation within clusters and used the MBN method to adjust for potential bias in the empirical sandwich estimator due to small numbers of patients with repeated measures. The outcome was modeled as a binary distribution using a logit link and accounting for clustering by study cluster and individual patient. We assumed a covariance structure of compound symmetry for correlation within clusters.

© 2024 Vinson DR et al. *JAMA Network Open.*

eResults. Intraclass Correlation and Churn Rates

We used mixed model regression methods, examining within and between cluster correlation over time to see if time-decay correlation might apply.[3-9] In this smaller subset of encounters where the patient was eligible for oral anticoagulation intervention, the inter-cluster correlation was 0.019 for the trial period, and correlations between clusters, study time period, and the outcome were all small ($P<0.05$) and not statistically significant (all P values >0.16).

The intra-cluster correlation coefficient was low at 0.025 for study clusters but very high for repeated patients, as expected (0.96 with 54 out of 1203 patients with more than 1 eligible encounter). Review of intra- and inter-cluster correlation by study month did not indicate any time trends, and so compound symmetry was chosen to account for clustering by patient and study cluster in the analysis.

As expected, churn in the open cohort was quite high, with only 4.5% of patients having more than one eligible encounter during the trial period. Churn ranged from a low of 89.2% in month 8 of the trial period to 100% in 4 out of 22 trial months.

With the cohort split between 9 clusters over 22 trial months, mixed models for monthly intraclass correlation calculations did not have positive Hessian matrices. Summarizing into 3 time periods of 7 months each, intraclass correlation for study clusters were all low: 0.006, 0.003, and 0.102 for beginning, middle and ending periods. Intraclass correlations for patient clustering in these same 3 study periods were 0.96, 0.99, and 0.90, respectively.

eTable 2. Emergency Department Patients with Primary Atrial Fibrillation or Atrial Flutter Eligible for Anticoagulation Initiation on Intervention Condition, Stratified by Oral Anticoagulation Initiation on Discharge or Within 30 Days

**Characteristics**	**Total (N=816)** n (%)	**Oral Anticoagulation Initiation** **Yes (n=558)** n (%)	**No (n=258)** n (%)	***P* value**
**Age, y**				
Mean (SD)	74.5 (10.3)	74.4 (9.2)	74.6 (12.2)	0.80
Median (IQR)	74.0 (68.0-81.0)	75.0 (69.0-81.0)	73.0 (67.0-84.0)	0.84
Range	38.0-101.0	39.0-101.0	38.0-99.0	
Missing	0 (0.0)	0 (0.0)	0 (0.0)	
Category				**0.04**
<65	109 (13.4)	63 (11.3)	46 (17.8)	
65-74	309 (37.9)	215 (38.5)	94 (36.4)	
≥75	398 (48.8)	280 (50.2)	118 (45.7)	
**Female**	406 (49.8)	278 (49.8)	128 (49.6)	0.96
**Male**	410 (50.2)	280 (50.2)	130 (50.4)	
**Race/ethnicity, self-reported**[a]				**0.03**
African American	60 (7.4)	36 (6.5)	24 (9.3)	
Asian	109 (13.4)	74 (13.3)	35 (13.6)	
Hispanic or Latinx	79 (9.7)	63 (11.3)	16 (6.2)	
White	533 (65.3)	356 (63.8)	177 (68.6)	
Other/Multi/Unknown[a]	35 (4.3)	29 (5.2)	6 (2.3)	
**Index atrial arrhythmia**				0.42
Atrial fibrillation	683 (83.7)	471 (84.4)	212 (82.2)	
Atrial flutter or both	133 (16.3)	87 (15.6)	46 (17.8)	
**Comorbidities**				
History of prior AFF	469 (57.5)	265 (47.5)	204 (79.1)	**<0.001**
Hypertension	677 (83.0)	469 (84.1)	208 (80.6)	0.23
Vascular disease	577 (70.7)	377 (67.6)	200 (77.5)	**0.004**
Diabetes	261 (32.0)	185 (33.2)	76 (29.5)	0.29
Congestive heart failure	113 (13.8)	55 (9.9)	58 (22.5)	**<0.001**
Ischemic stroke, transient ischemic attack, or thromboembolic disease	72 (8.8)	43 (7.7)	29 (11.2)	0.10
**CHA_2DS_2-VASc Score**				
Mean (SD)	4.0 (1.5)	4.0 (1.4)	4.1 (1.7)	0.35
Median (IQR)	4.0 (3.0-5.0)	4.0 (3.0-5.0)	4.0 (3.0-5.0)	0.87
Range	2.0-9.0	2.0-9.0	2.0-9.0	

© 2024 Vinson DR et al. *JAMA Network Open.*

eTable 2. Emergency Department Patients with Primary Atrial Fibrillation or Atrial Flutter Eligible for Anticoagulation Initiation on Intervention Condition, Stratified by Oral Anticoagulation Initiation on Discharge or Within 30 Days

Characteristics	Total (N=816) n (%)	Oral Anticoagulation Initiation: Yes (n=558) n (%)	Oral Anticoagulation Initiation: No (n=258) n (%)	*P* value
Missing	0 (0.0)	0 (0.0)	0 (0.0)	
Category				**0.001**
2-3	345 (42.3)	229 (41.0)	116 (45.0)	
4-5	334 (40.9)	248 (44.4)	86 (33.3)	
≥6	137 (16.8)	81 (14.5)	56 (21.7)	
**Decision Support Use**				**0.008**
Yes	217 (26.6)	164 (29.4)	53 (20.5)	
No	599 (73.4)	394 (70.6)	205 (79.5)	

AFF, atrial fibrillation or atrial flutter.
[a]Other race/ethnicity includes Native American and Hawaii and Pacific Islander.

P values bold if <0.05.

eTable 3. Emergency Department Patients With Primary Atrial Fibrillation or Atrial Flutter Eligible for Anticoagulation Initiation During Intervention Phase, Stratified by Clinical Decision Support Use

Characteristics	Total (N=816) n (%)	Clinical Decision Support Use: Yes (n=217) n (%)	Clinical Decision Support Use: No (n=599) n (%)	*P* value
**Age, y**				
Mean (SD)	74.5 (10.3)	73.5 (10.2)	74.8 (10.3)	0.11
Median (IQR)	74.0 (68.0-81.0)	74.0 (68.0-79.0)	75.0 (68.0-82.0)	0.20
Range	38.0-101.0	39.0-99.0	38.0-101.0	
Missing	0 (0.0)	0 (0.0)	0 (0.0)	
Category				0.34
<65	109 (13.4)	33 (15.2)	76 (12.7)	
65-74	309 (37.9)	87 (40.1)	222 (37.1)	
≥75	398 (48.8)	97 (44.7)	301 (50.3)	
**Female**	406 (49.8)	100 (46.1)	306 (51.1)	0.21
**Male**	410 (50.2)	117 (53.9)	293 (48.9)	
**Race/ethnicity, self-reported**[a]				0.76
African American	60 (7.4)	16 (7.4)	44 (7.3)	
Asian	109 (13.4)	31 (14.3)	78 (13.0)	

© 2024 Vinson DR et al. *JAMA Network Open.*

eTable 3. Emergency Department Patients With Primary Atrial Fibrillation or Atrial Flutter Eligible for Anticoagulation Initiation During Intervention Phase, Stratified by Clinical Decision Support Use

Characteristics	Total (N=816) n (%)	Clinical Decision Support Use Yes (n=217) n (%)	No (n=599) n (%)	*P* value
Hispanic or Latinx	79 (9.7)	18 (8.3)	61 (10.2)	
White	533 (65.3)	140 (64.5)	393 (65.6)	
Other/Multi/Unknown[a]	35 (4.3)	12 (5.5)	23 (3.8)	
**Index atrial arrhythmia**				0.73
Atrial fibrillation	683 (83.7)	180 (82.9)	503 (84.0)	
Atrial flutter or both	133 (16.3)	37 (17.1)	96 (16.0)	
**Comorbidities**				
History of prior AFF	469 (57.5)	106 (48.8)	363 (60.6)	**0.003**
Hypertension	677 (83.0)	179 (82.5)	498 (83.1)	0.83
Vascular disease	577 (70.7)	147 (67.7)	430 (71.8)	0.26
Diabetes	261 (32.0)	70 (32.3)	191 (31.9)	0.92
Congestive heart failure	113 (13.8)	27 (12.4)	86 (14.4)	0.48
Ischemic stroke, transient ischemic attack, or thromboembolic disease	72 (8.8)	21 (9.7)	51 (8.5)	0.85
**CHA_2DS_2-VASc Score**				
Mean (SD)	4.0 (1.5)	3.9 (1.5)	4.1 (1.5)	0.16
Median (IQR)	4.0 (3.0-5.0)	4.0 (3.0-5.0)	4.0 (3.0-5.0)	0.17
Range	2.0-9.0	2.0-8.0	2.0-9.0	
Missing	0 (0.0)	0 (0.0)	0 (0.0)	
Category				0.15
2-3	345 (42.3)	90 (41.5)	255 (42.6)	
4-5	334 (40.9)	98 (45.2)	236 (39.4)	
≥6	137 (16.8)	29 (13.4)	108 (18.0)	

AFF, atrial fibrillation or atrial flutter.
[a]Other race/ethnicity includes Native American and Hawaii and Pacific Islander

P values bold if <0.05.

© 2024 Vinson DR et al. *JAMA Network Open.*

eDiscussion 1. Results in the Context of Our Power Calculations

The power calculations for this trial estimated 63 anticoagulation- eligible patients per month or 1,386 patients for the entire study period. Additional power calculation assumptions included a churn rate of 0.984, inter cluster correlation (ICC) of 0.006, cluster autocorrelation of 0.356, and an individual autocorrelation coefficient of zero assuming a discrete-time decay. Our power calculation estimated that we could identify a 4.9% increase in OAC action taken with 80% power.

During the study, only 1,203 OAC-eligible patients were identified —87% of what was anticipated, thus reducing power to detect our targeted difference. Our assumption of a low ICC was reasonable, with the ICC for the study period being slightly higher at 0.025, potentially reducing power slightly. However, our assumed cluster autocorrelation was incorrect. We checked for autocorrelation in each cluster using an autoregression model and found no evidence of autocorrelation within any of the study clusters during the study period. This lack of autocorrelation likely reduced power to detect a difference as the actual autocorrelation could not increase the precision of our estimate. We assumed an individual autocorrelation of zero and very high churn, with few patients contributing multiple measures in this open cohort. Our actual churn rate of 0.955 was slightly lower than anticipated, and the individual autocorrelation was quite high at 0.96, among the 54 patients with multiple measures. The high individual autocorrelation should increase power to detect a difference, but only slightly. Overall, we expect that the combination of these factors decreased our power to detect a difference of 4.9%, and so, although we identified a 5.4% increase, it failed to reach statistical significance (P=0.07 unadjusted, P=0.13 adjusted).

We also performed a sensitivity analysis using only the first eligible encounter for each patient to remove the impact of individual autocorrelation and the resulting estimate changed only minimally and still did not reach statistical significance.

eDiscussion 2. Pre-to-Post Studies of Anticoagulation-Focused Decision Support Interventions in the Emergency Department

Several pre-post studies of OAC-focused CDSS interventions in the ED have demonstrated improvements in OAC initiation among eligible patients with AF at and following discharge.[11-13] Comparisons between these studies and the O'CAFÉ trial are complicated by significant differences in study design (e.g., pre-post with lack of controls[12,13] or controls without randomization[11]), setting (e.g., university vs community), anticoagulation eligibility criteria (e.g., excluding patients >80 years,[12] who were included in O'CAFÉ), baseline ED OAC initiation rates (e.g., 15%[12] vs 49% in O'CAFÉ), the nature of interventions (e.g., providing post-ED follow-up in cardiology clinic[11-12]), and timing of OAC ascertainment (e.g., nothing beyond the ED[13] or 90 days beyond the ED[11]).

© 2024 Vinson DR et al. *JAMA Network Open.*

eReferences

1. Vinson DR, Rauchwerger AS, Karadi CA, et al. Clinical decision support to Optimize Care of patients with Atrial Fibrillation or flutter in the Emergency department: protocol of a stepped-wedge cluster randomized pragmatic trial (O'CAFÉ trial). *Trials*. 2023;24(1):246. doi:10.1186/s13063-023-07230-2
2. January CT, Wann LS, Calkins H, et al. 2019 AHA/ACC/HRS Focused Update of the 2014 AHA/ACC/HRS Guideline for the Management of Patients With Atrial Fibrillation: A Report of the American College of Cardiology/American Heart Association Task Force on Clinical Practice Guidelines and the Heart Rhythm Society. *J Am Coll Cardiol*. 2019;74(1):104-132. doi:10.1016/j.jacc.2019.01.011
3. National Institutes of Health: Research Methods Resources: Stepped Wedge Group-Randomized Trials 2022. https://researchmethodsresources.nih.gov/methods/SWGRT Accessed August 4, 2024.
4. National Institutes of Health. Research Methods Resources: Stepped Wedge Group-Randomized Trial Sample Size Calculator https://researchmethodsresources.nih.gov/Calculators#swgrt Accessed August 30, 2024.
5. Kasza J, Hooper R, Copas A, Forbes AB. Sample size and power calculations for open cohort longitudinal cluster randomized trials. *Stat Med.* 2020;39(13):1871-1883..
6. Hemming K, Kasza J, Hooper R, et al. A tutorial on sample size calculation for multiple-period cluster randomized parallel, cross-over and stepped-wedge trials using the Shiny CRT Calculator. *Int J Epidemiol*. 2020;49(3):979-995.
7. Kasza J, Forbes AB. Inference for the treatment effect in multiple-period cluster randomised trials when random effect correlation structure is misspecified. *Stat Methods Med Res*. 2019;28(10-11):3112-3122.
8. Kasza J, Hemming K, Hooper R, Matthews JNS, Forbes AB. Impact of non-uniform correlation structure on sample size and power in multiple-period cluster randomised trials. *Stat Methods Med Res*. 2019;28(3):703-716.
9. Hooper R, Teerenstra S, de Hoop E, et al. Sample size calculation for stepped wedge and other longitudinal cluster randomised trials. *Stat Med*. 2016;35(26):4718-4728.
10. SAS Help Center. The GLIMMIX Procedure. Example 51.12 Fitting a Marginal (GEE-Type) Model. https://documentation.sas.com/doc/en/statug/15.2/statug_glimmix_examples15.htm Accessed August 30, 2024.
11. Rezazadeh S, Chew DS, Miller RJH, et al. Effects of a reminder to initiate oral anticoagulation in patients with atrial fibrillation/atrial flutter discharged from the emergency department: REMINDER study. *CJEM*. 2018;20(6):841-849. doi:10.1017/cem.2018.415
12. Parkash R, Magee K, McMullen M, et al. The Canadian Community Utilization of Stroke Prevention Study in Atrial Fibrillation in the Emergency Department (C-CUSP ED). *Ann Emerg Med*. 2019;73(4):382-392. doi:10.1016/j.annemergmed.2018.09.001
13. Barbic D, DeWitt C, Harris D, et al. Implementation of an emergency department atrial fibrillation and flutter pathway improves rates of appropriate anticoagulation, reduces length of stay and thirty-day revisit rates for congestive heart failure. *CJEM*. 2018;20(3):392-400. doi:10.1017/cem.2017.418

© 2024 Vinson DR et al. *JAMA Network Open.*
