# Supplementary material for: Decision Support Intervention and Anticoagulation for Emergency Department Atrial Fibrillation: The O’CAFÉ Stepped-Wedge Cluster Randomized Clinical Trial
Source: JAMA Netw Open. 2024 Nov 6;7(11):e2443097. doi: 10.1001/jamanetworkopen.2024.43097 (PMC11541643; doi:10.1001/jamanetworkopen.2024.43097)
Supplement: Supplement 2. — Trial Protocol [file jamanetwopen-e2443097-s002.pdf]

# Study Protocol

**Title (short):** Clinical Decision Support for Atrial Fibrillation and Flutter

**Title (official):** Reducing Variation in Hospitalization and Processes of Care in Emergency Department Patients with Atrial Fibrillation: A Stepped Wedge Cluster Randomized Trial

**Principal Investigator:** David R. Vinson, MD, Senior Physician, The Permanente Medical Group; Co-chair, CREST Network; and Adjunct Investigator, KP Division of Research

**Lead Co-Investigators:** Mary E. Reed, DrPH, and Dustin G. Mark, MD

**Administrator**: Adina Rauchwerger, MPH

**Trial Registration**: https://clinicaltrials.gov/study/NCT05009225

**Date:** August 01, 2021

**Brief Summary**

Atrial fibrillation (AF) is a major public health problem: it impairs quality of life and independently heightens the risks of ischemic stroke, heart failure and all-cause mortality. AF is a common reason for presenting to emergency departments (ED) in Kaiser Permanente Northern California (KPNC) and is associated with frequent hospitalization. Additionally, inter-facility hospitalization rates for AF vary across KPNC. Improvements in modifiable components of ED AF care could potentially reduce low-yield hospitalizations and the associated costs, patient inconveniences, and complications that can ensue. Real-time clinical decision support systems (CDSS) can transform entrenched physician practices and improve patient outcomes. The investigators will conduct a stepped-wedge cluster randomized trial of a CDSS intervention across 13 KPNC EDs for the comprehensive management of acute AF with the following 2 aims: 1) To evaluate the impact of the CDSS intervention on index hospitalization rates (as well as on ED AF rate and rhythm control process-of-care metrics); 2) To evaluate the impact of the CDSS intervention on AF stroke prevention actions for eligible participants at the time of ED discharge and in the following 30 days. The investigators hypothesize that the CDSS intervention will safely reduce index hospitalization rates, improve rate and rhythm control process-of-care metrics, and increase stroke prevention actions for eligible participants at ED discharge and within 30 days.

**BACKGROUND**

Atrial fibrillation (AF) and atrial flutter are prevalent in the United States and are likely to escalate as the population continues to age. These atrial arrhythmias have a substantial impact on quality of life and patient health, increasing the risk for heart failure, thromboembolism, hospitalization, and death. The economic burden on the health care system is considerable.[1, 2]

Patients with symptomatic AF and atrial flutter often present to the emergency department (ED) for treatment. There is no definitive evidence supporting optimal ED management of patients with AF and atrial flutter. Treatment strategies vary widely between countries, within countries, and within facilities.[3-8] Not all of this variation is warranted.[9] Implementation of professional society-based guidelines may help standardize care around best practices. But professional society-based guidelines for AF treatment vary in the amount of attention given to emergency medicine-related issues and offer variable recommendations for acute management.[10-12]

Using recommendations from various clinical practice guidelines, as well as from primary studies and internal best practices, we created a set of recommendations for emergency medicine physicians, addressing 3 leading aspects of ED care: (1) achieving sustained rate reduction for patients with rapid ventricular response; (2) optimizing cardioversion by increasing first shock success or using suitable pharmacologic agents; (3) increasing implementation of stroke prevention actions in eligible patients being discharged home (**Table 1**). By improving rate reduction and cardioversion we sought to reduce hospitalization, at least in medical centers with higher hospitalization rates.[13] By promoting stroke prevention actions, we sought to increase the 30-day incidence of anticoagulation initiation for eligible patients.

**Table 1**. Management recommendations to improve care of ED patients with atrial fibrillation and flutter.

| **Major Recommendations in Electronic Clinical Decision Support Application*** | **Rationale for Recommendation** |
|---|---|
| **1. Sustained rate reduction** | |
| Administer long-acting rate-reducing medications early in the ED encounter, either in addition to or in lieu of standard intravenous (IV) bolus medications | Medications with sustained effect on rapid ventricular response have been central to multifaceted ED interventions associated with reduced hospitalization of patients with primary AF or atrial flutter.[14, 15] |
| **2. Effective cardioversion** | |
| **2A. Electrical** | |
| Start with maximal joules and consider | These measures improve first-shock success |

| | |
|---|---|
| manual pressure augmentation, especially for obese patients | and may reduce sedation duration and risk.[11, 16, 17] |
| **2B. Pharmacologic** | |
| Consider efficiency in addition to effectiveness, safety, and ease of administration when selecting medications | For example, medications with a shorter time to effect, e.g., IV procainamide[18] (median 30-40 min), facilitate ED operational efficiencies, unlike IV amiodarone, which does not distinguish itself from placebo for 6-8 hours.[19] |
| **3. Stroke prevention** | |
| A. Identify patients at risk using auto-populating validated scoring system | Stroke risk stratification is the essential preparatory step for any subsequent stroke prevention action.[11, 12, 20, 21] |
| B. Print risk-specific handout for eligible patients and review with patient and family at bedside | The handout helps initiate a shared decision-making conversation on stroke prevention[22] that can continue with outpatient physicians following discharge to home. |
| C1. Initiate outpatient anticoagulation at the time of ED discharge to home | Oral anticoagulation with DOACs or warfarin significantly reduces ischemic stroke and death in patients with AF or atrial flutter. Prescription on ED discharge can be associated with higher long-term use than when prescribing is left to post-discharge outpatient care.[23, 24] |
| C2. Or electronically consult the Anticoagulation Management Service to request they contact patients who want to learn more about stroke prevention before initiating anticoagulation | Following discharge to home, anticoagulation pharmacists can call eligible patients to provide in-depth education on benefits and risks of anticoagulation for stroke prevention.[25, 26] |
| **4. Timely Follow-up** | |
| a. Encourage or request close follow-up (<7d) with outpatient physicians | Transferring care to outpatient physicians who can oversee longitudinal care of AF and atrial flutter and related conditions is key to |

| | |
|---|---|
| | long-term management success.[12] Moreover, follow-up of these patients within a week of discharge has been associated with a reduction in the rate of death and hospitalization within 1 year.[27] |

AF = atrial fibrillation; DOAC, direct oral anticoagulant; ED = emergency department
* RISTRA-AF also reminds physicians to inquire of their AF and atrial flutter patients about 2 dietary triggers: cold drink/food and alcohol.[28]

With the goal of making our treatment recommendations readily available to physicians at the point of care, we designed a web-based clinical decision support system (CDSS), called RISTRA-AF (RISTRA stands for Risk Stratification). This decision-support application, similar to prior RISTRA applications, is embedded within the ED navigator of the electronic health record (EHR).[29, 30] We recently completed a 3-center pilot study to evaluate the feasibility and user response of the CDSS, which allowed us to improve RISTRA-AF. In what follows, we describe the pragmatic stepped-wedge cluster randomized trial.

**Objectives**

We have 2 primary aims:

1. To reduce initial hospitalization for adults (≥18 years) presenting to the ED with primary AF or atrial flutter.

   We hypothesize that implementation of RISTRA-AF will reduce initial hospitalization for ED adults (≥18 years) with primary AF and atrial flutter.

2. To increase the proportion of ED adult health plan members with primary AF or atrial flutter eligible for anticoagulation being discharged home who are prescribed anticoagulation either at the time of discharge or within the following 30 days.

   We hypothesize that implementation of RISTRA-AF will increase the proportion of ED adult health plan members eligible for anticoagulation initiation on discharge to home who are prescribed anticoagulation at the time of discharge or within the following 30 days.

## METHODS

**Trial Design**

This stepped-wedge cluster randomized pragmatic superiority trial will be undertaken across 13 EDs in a large, integrated healthcare delivery system in the United States. Trials EDs were selected by (a) having an on-site study champion (a clinical peer of the department and a co-investigator with the CREST research network; n=16) and (b) having not already participated in the pilot study (n=3). Though not without shortcomings, this design was selected over a parallel group design for 3 reasons:[31] (1) The educational program of a staggered rollout can be easier to implement than the alternative of a traditional parallel group design. With only 1 cluster launching each month,

the principal investigator will be able to co-present with site leads when introducing study material to their emergency and ancillary departments (i.e., adult hospital medicine and cardiology). This would be infeasible if multiple clusters launched simultaneously. (2) This design expands intervention exposure across all study EDs, which is desirable as the intervention is thought to be an improvement over usual care. (3) This approach maximizes power because the intervention effect is estimated not only by between-cluster comparisons but also by within-cluster comparisons. We designed this as a pragmatic trial in which the intervention could be tested under conditions closer to usual care than ideal care.[32]

Among the 13 study EDs, 8 will function as 4 operational dyads, pairs of EDs, each served by 1 shared staff of emergency physicians. Keeping these EDs paired, we have 9 study clusters, which the principal investigator has allocated to 1 of 9 sequences using a computer-generated randomization sequence. Site leads were not blinded to their launch month as they need to schedule educational presentations. Physicians in study EDs could not be blinded to interventions; patients, however, were unaware of the trial. After an initial period of 3 months in which all clusters will be in the control condition (July through September 2021), the intervention will be implemented in 1 cluster per step at 1-month intervals (**Figure 1**). The first 2 months of implementation will serve as a transition period, which will not be analyzed. The staggered roll-out will occur over 9 months (October 2021 through June 2022), after which all clusters will be in the transition or intervention condition. The total study duration is planned for 22 months, completing enrollment April 30, 2023.

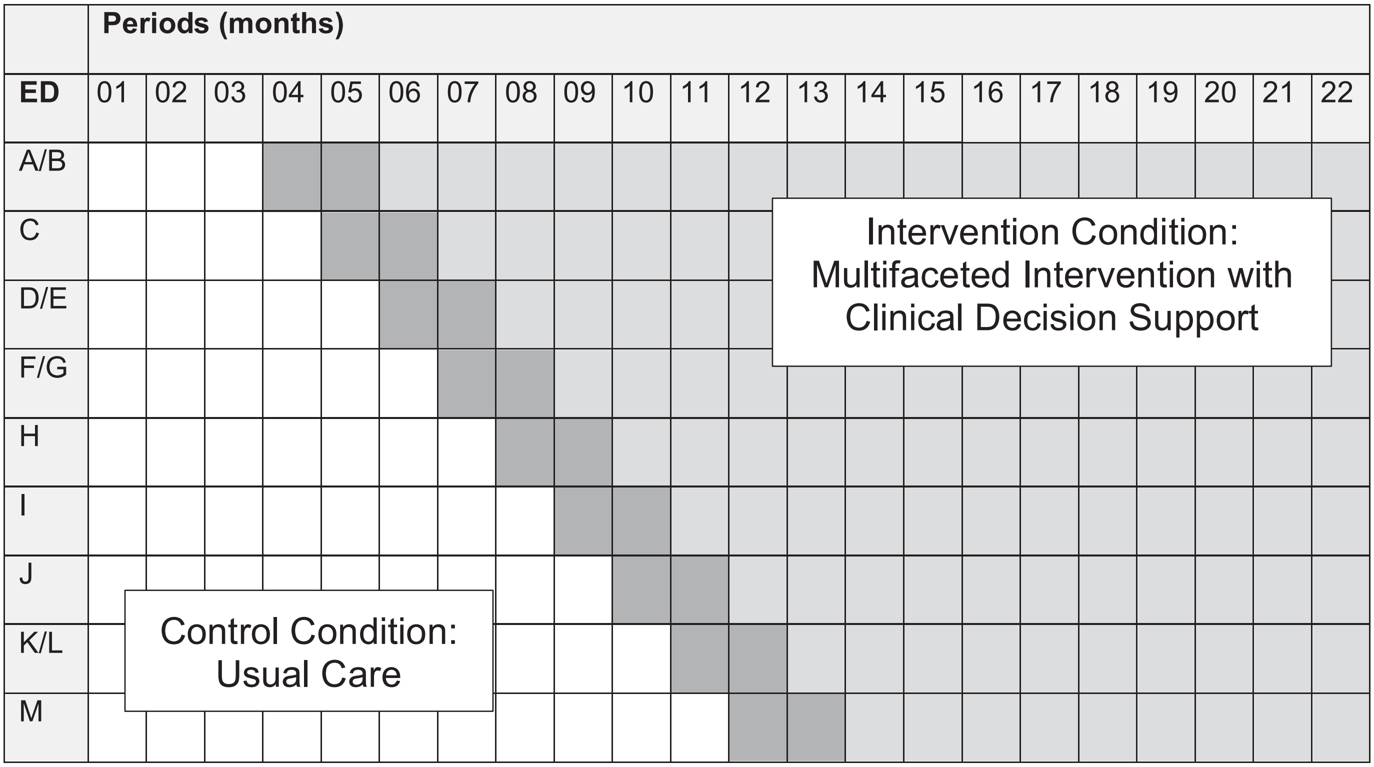


**Figure 1**. Time course over which 13 emergency departments (EDs) (labeled A-M) crossed over from control to intervention condition.

**Study setting**

The trial will be conducted in EDs of community medical centers in Kaiser Permanente (KP) Northern California, a large U.S. integrated health system that provides comprehensive inpatient and outpatient care for more than 4.5 million members. Health plan members include over 33% of the population in areas served and are highly representative of the ethnic and socioeconomic diversity of the surrounding and statewide population.[33] Sixteen of the 21 EDs of KP Northern California have on-site emergency physicians who are embedded researchers and clinical investigators with the KP CREST Network. They serve as site leads for pragmatic trials, providing necessary on-the-ground study promotion, physician education and feedback among their peers.[29] Three of these 16 EDs are participating in the pilot study and are ineligible for the pragmatic trial. The remaining 13 EDs have agreed to participate in the pragmatic trial.

KP Northern California is a learning health care system with a strategic delivery science agenda[34] and is supported by a comprehensive, integrated EHR that includes inpatient, outpatient, emergency, pharmacy, laboratory, and imaging data.[35] Six of the 13 study EDs participate to some degree in resident training. Patient care decisions are at the discretion of the treating physicians. No departmental policies or scripted pathways are in place for ED rate reduction or cardioversion of patients with AF or atrial flutter. In prior studies we had observed significant inter-facility variation in ED AF management.[13] Treating physicians have access to the standard KP Northern California discharge order-set for AF-related stroke prevention, which currently recommends dabigatran, a direct oral anticoagulant, as first-line thromboprophylaxis for eligible patients. Outpatient anticoagulation with both warfarin and direct oral anticoagulants is managed closely by a pharmacy-led, telephone-based Anticoagulation Management Service.[36] All emergency physicians have around-the-clock access to on-call cardiology consultants.

**Study Participants and Study Patients**

Study participants will include emergency physicians working in the 13 study EDs during the study period, all of whom are board-certified (or board-eligible) emergency physicians. A small proportion (<5%) of emergency physicians are part-time moonlighters.

Study patients will be adults (≥18 years) receiving care for primary AF or atrial flutter (using ICD codes) in a participating ED, regardless of whether the RISTRA-AF application is employed. We will exclude patients from RISTRA AF and from the trial for any of the following concurrent ED diagnoses: pregnancy, ST-elevation myocardial infarction, acute myo- or pericarditis, acute pneumonia, pulmonary embolism, shock (e.g., septic, hemorrhagic, cardiogenic), recent major thoracic trauma (<48h), thyroid storm, or acute toxidrome (e.g., sympathomimetic or anticholinergic). We chose not to exclude heart failure co-diagnoses, as we want to provide treatment recommendations for patients with AF and atrial flutter and co-existing heart failure.

**Multifaceted Intervention**

In addition to CDSS access, the intervention phase will include physician education, monthly study

promotion, and eventual facility-specific audit and feedback. Physician education will begin with each facility's transition to the intervention phase and will address AF management recommendations (Table 1) and use of RISTRA-AF. Local site leads will thereafter provide their EDs with monthly emails. The content will include commendation to recent local RISTRA-AF users, highlights of overall study progress, and "test your knowledge" questions to keep the AF and atrial flutter education going beyond the initial training episode. Six months following launch at a study site, site leads will present at an ED meeting a brief overview of RISTRA-AF. We will create an automatic audit and feedback tool to display intra-facility comparisons on the following metrics: use of long-acting rate reduction medications and anticoagulation prescribing at time of ED discharge to home and in the 30 days following discharge. This will roll-out after several EDs have entered the intervention phase and comparisons can be undertaken.

**Clinical Decision Support System**

We made our management recommendations readily accessible to emergency physicians in the pilot study by transposing them into an established web-based CDSS (RISTRA). We followed CDSS design principles that have been shown effective in our setting in earlier applications.[37, 38] The RISTRA system is used to provide point-of-care decision support to help emergency physicians in the management of adults with acute pulmonary embolism,[29] adults with chest pain,[30] children 5 years of age or greater with acute abdominal pain,[38, 39] febrile infants, and syncope/presyncope. RISTRA is accessed by a hyperlink button that was added to our ED Navigator of the EHR (Epic, Verona, Wisconsin) and seamlessly fits within the flow of patient care.[38, 40]

We summarized the treatment recommendations above in **Table 1** and detail them in what follows.

**Common Ingestion Triggers**

Though the ED is commonly the place where patients with acutely symptomatic AF and atrial flutter seek medical care, little attention in emergency medicine has been paid to the clinician's role in helping patients identify and manage reversible triggers of paroxysmal AF and atrial flutter. To redress this oversight, we designed RISTRA-AF to prompt physicians to ask about 2 widespread ingestion triggers: cold drink/food and alcohol. Cold drink and food can precipitate AF and atrial flutter within seconds or minutes of ingestion.[28, 41] Some physicians are unaware of the causal connection between cold drink/food and AF and atrial flutter and have been known to dismiss their patient's trigger claims.[28] Alcohol binging is a well-known cause of AF and atrial flutter, known as "Holiday Heart."[42-44]

When clinician-directed inquiries about these 2 triggers elicit a positive response, the stage is set for patient behavioral changes that may have remarkable benefits by decreasing the overall AF burden.[28, 45] Reducing recurrent events can reduce the attending symptoms, risk, and inconvenience of episodic AF and atrial flutter, as well as the costs incurred when the recurrence leads to missed work and the need for urgent medical care. We chose not to include coffee

consumption among our list of triggers because the evidence does not support the commonly held belief that coffee triggers AF and atrial flutter.[46]

**Modular Approach**

After addressing common AF triggers and assisting in populating the CHADS-VASc score (more on this below), RISTRA-AF brought physician users to the main modules screen. Here users can readily access recommendations on rate control, cardioversion, and stroke prevention (**Figure 2**).

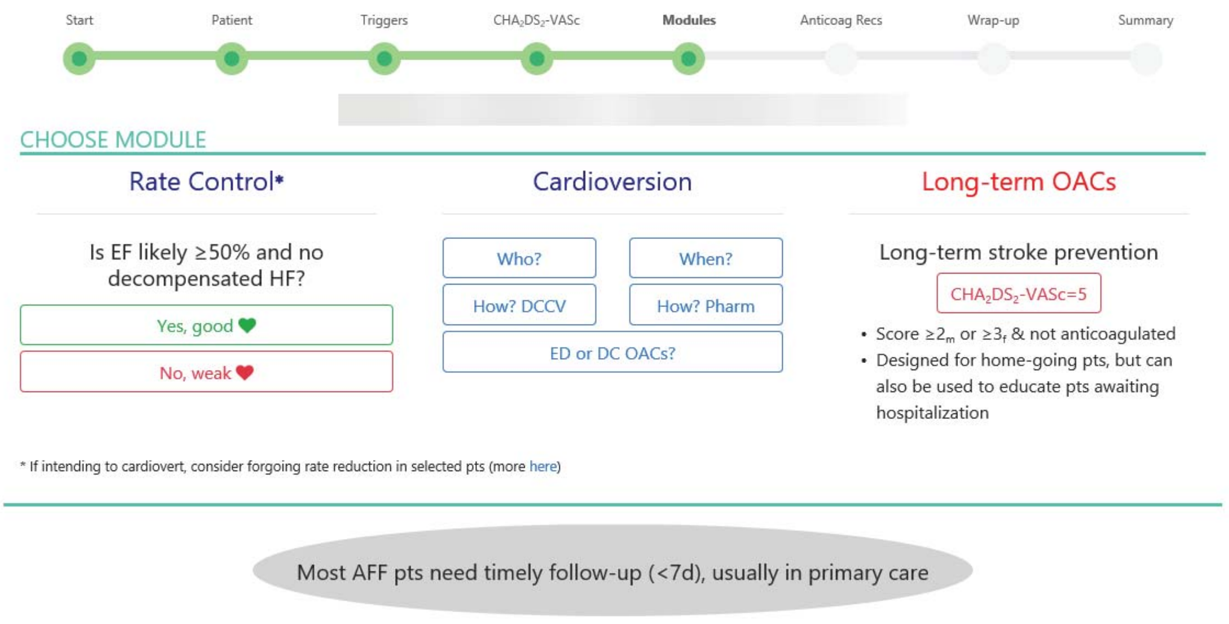


**Figure 2**. Modules screen of the RISTRA-AF clinical decision support system
EF, ejection fraction; HF, heart failure; OAC, oral anticoagulation; pts, patients; ♥, heart

**Rate Reduction**

Slowing rapid ventricular response is the most common treatment emergency physicians provide their patients with AF and atrial flutter. IV medications, like the non-dihydropyridine calcium channel blocker diltiazem and the beta-adrenergic receptor blocker metoprolol, are effective heart rate-reducing medications (rate reducers) with a rapid onset.[47, 48] Unfortunately, bolus doses of IV rate reducers can have a relatively short duration of action. The effect of a single bolus of IV diltiazem, for example, wanes after 1-3 hours. If the rapid ventricular response returns, it can rebound higher than the initial rate. This may prompt another IV bolus of rate-reducing medication. If the rapid ventricular response again recurs, a continuous infusion of diltiazem may follow, or alternative IV rate control medications, which may occasion admission to an observation unit or hospital ward for continued heart rate management.

One strategy to avoid this common route to protracted care is the early administration of oral long-acting rate reducers, e.g., diltiazem XR or metoprolol tartrate. These can be given in addition to (or in lieu of) their IV counterparts.[20] The combination of shorter-acting IV medications with

longer-acting medications has the advantage of providing both immediate and sustained rate-reducing effects. Several studies in different U.S. ED settings have found that treatment pathways encouraging early administration of a long-acting oral rate-reducing medication (with or without a concomitant IV rate reducer) decrease hospitalization of stable patients with primary primary AF.[14, 15] IV magnesium sulfate is another effective rate reducer, which can be helpful independent of a patient's serum magnesium level.[49-51] Studies have shown continued effect lasting 12-24 hours following initial magnesium sulfate administration.[49, 51, 52] Early administration of these "sustainers" (long-acting oral medications or IV magnesium sulfate) may reduce the need for hospitalization. We recommend "sustainers" only for normotensive patients with a "good heart," defined as 1 with an ejection fraction greater than 50% (based on recent echocardiography or physician gestalt) and no clinical evidence of decompensated heart failure (**Figure 3**).

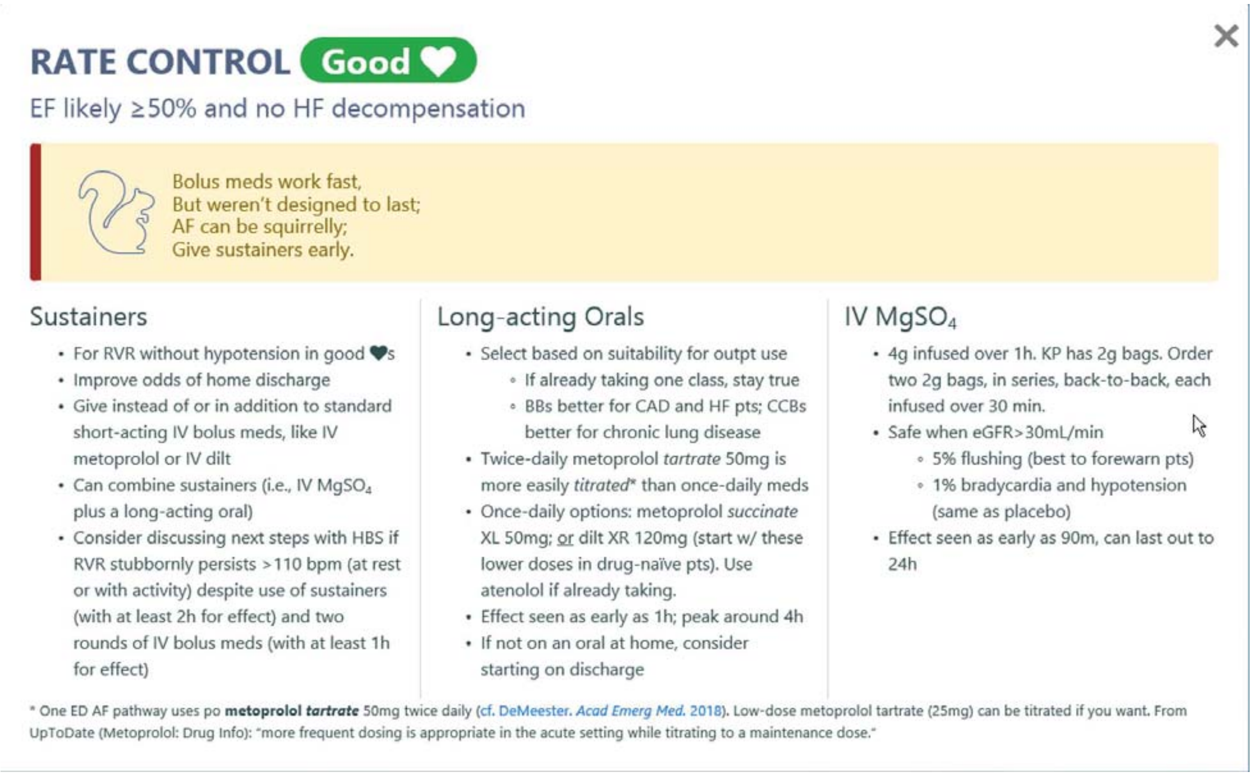


**Figure. 3**. The rate control screen in RISTRA-AF for patients with a "good heart".
A good heart is defined as 1 with an ejection fraction greater than 50% (based on recent echocardiography or physician gestalt) and no clinical evidence of decompensated heart failure.
BB, beta-blocker; bpm, beats per minute; CAD, coronary artery disease; CCB, calcium channel blocker; dilt, diltiazem; EF, ejection fraction; eGFR, estimated glomerular filtration rate; HBS, hospital-based specialist in internal medicine; HF, heart failure; IV, intravenous; KP, Kaiser Permanente; outpt, outpatient; Pharm, pharmacologic cardioversion; pt, patient; RVR, rapid ventricular response; w/, with.

Rate reduction in patients with hypotension, known left ventricular ejection fraction ≤50%, or decompensated heart failure is more challenging and warrants a different set of recommendations. If the physician is intent on attempting cardioversion in the ED and the stable patient is tolerating rapid ventricular response, we recommend against rate-reducing medications,

as some evidence suggests they may reduce the effectiveness of electrical cardioversion.[53] This does not apply to patients who are to receive oral flecainide or propafenone, as they require a rate-reducing agent to block the atrioventricular node at least 30 minutes prior to cardioversion.[54]

**Cardioversion**

Restoration of sinus rhythm is the most effective means of symptom resolution in patients with intermittent AF and atrial flutter and can be 1 component of a larger, long-term rhythm control strategy. Among ED patients, elective cardioversion is associated with reduced hospitalization and greater patient satisfaction.[18, 55, 56] RISTRA-AF provides recommendations about which ED patients may be candidates for elective and emergent cardioversion.[11] RISTRA-AF reminds physicians of the pros and cons to immediate attempted cardioversion compared with a short-term delay for those with symptomatic AF or atrial flutter of presumed recent-onset (<48 hours). The delayed approach is a “wait and see” approach that involves discharging the patient to home with a scheduled return visit at approximately 40 hours post-symptom onset. We leave the timing debate (today vs tomorrow) open to accommodate physician and patient preference as well as varied local practice patterns.[57-61] RISTRA-AF summarizes recommendations from varied sources about which patients are thought safe to cardiovert without several weeks of preceding anticoagulation and which patients may benefit from anticoagulation following ED cardioversion.[11, 54, 62-65]

**Electrical Cardioversion: Increasing First-shock Success**

When physicians elect to pursue ED cardioversion, we provide recommendations in RISTRA-AF to facilitate timely and effective sinus restoration (**Figure 4**). With synchronized electrical cardioversion, we recommend maximizing joules to optimize first-shock success and limit sedation time and risk.[17, 54] We recommend starting with maximal joules, which at present in our EDs is 200 (biphasic). If the first shock fails, a second shock can be administered at 1 minute. We recommend manual pressure augmentation to reduce transthoracic impedance, deliver more current to the heart and increase effectiveness of electrical cardioversion.[16, 66, 67] Manual pressure augmentation has been shown to be safe for the proceduralist.[16] It can be helpful for all patients, but more so for obese patients, who fail electrical cardioversion at twice the rate of non-obese patients.[16]

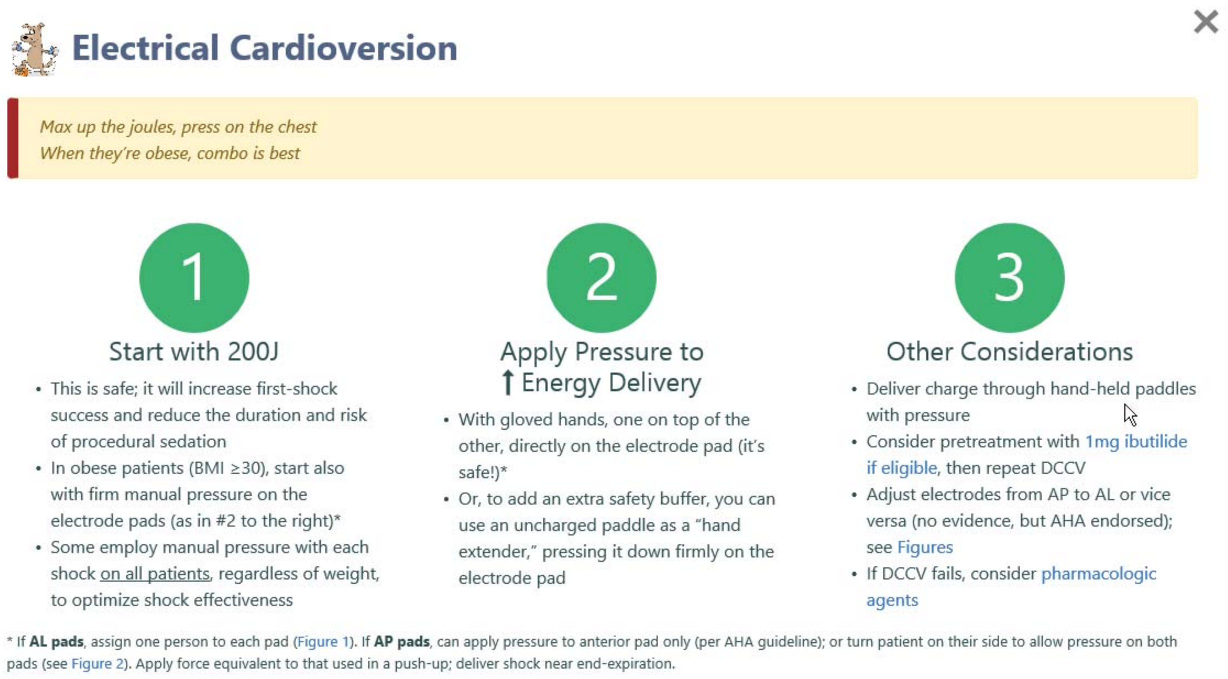


**Figure 4**. Electrical cardioversion screen in RISTRA-AF
AHA, American Heart Association; AL, anterior lateral; AP, anterior posterior; BMI, body mass index; DCCV, direct current cardioversion, max, maximize.

If the first 2 shocks with maximal joules and manual pressure augmentation are unsuccessful, a priming dose of ibutilide (1 mg over 10 minutes) can be used in eligible patients (the criteria are spelled out in RISTRA-AF), followed by another attempt at electrical cardioversion. This has been shown to increase sinus restoration.[54, 68] In response to failed electrical cardioversion, RISTRA-AF follows U.S. guidelines in suggesting changing pad placement from anterior-posterior to anterior-lateral or vice versa.[54] One can also switch to pharmacologic approaches.

**Time-efficient Pharmacologic Cardioversion**

Pharmacologic cardioversion is less effective than an electrical approach.[18] However, it may be preferred when patients are poor sedation candidates or refuse electrical cardioversion, if ED nursing staff cannot easily support elective procedural sedation, or if physicians (or departments) prefer a 2-step approach, starting with the less resource intensive pharmacotherapy and reserving sedation and synchronized cardioversion for those who fail step 1 (**Figure 5**).[69, 70]

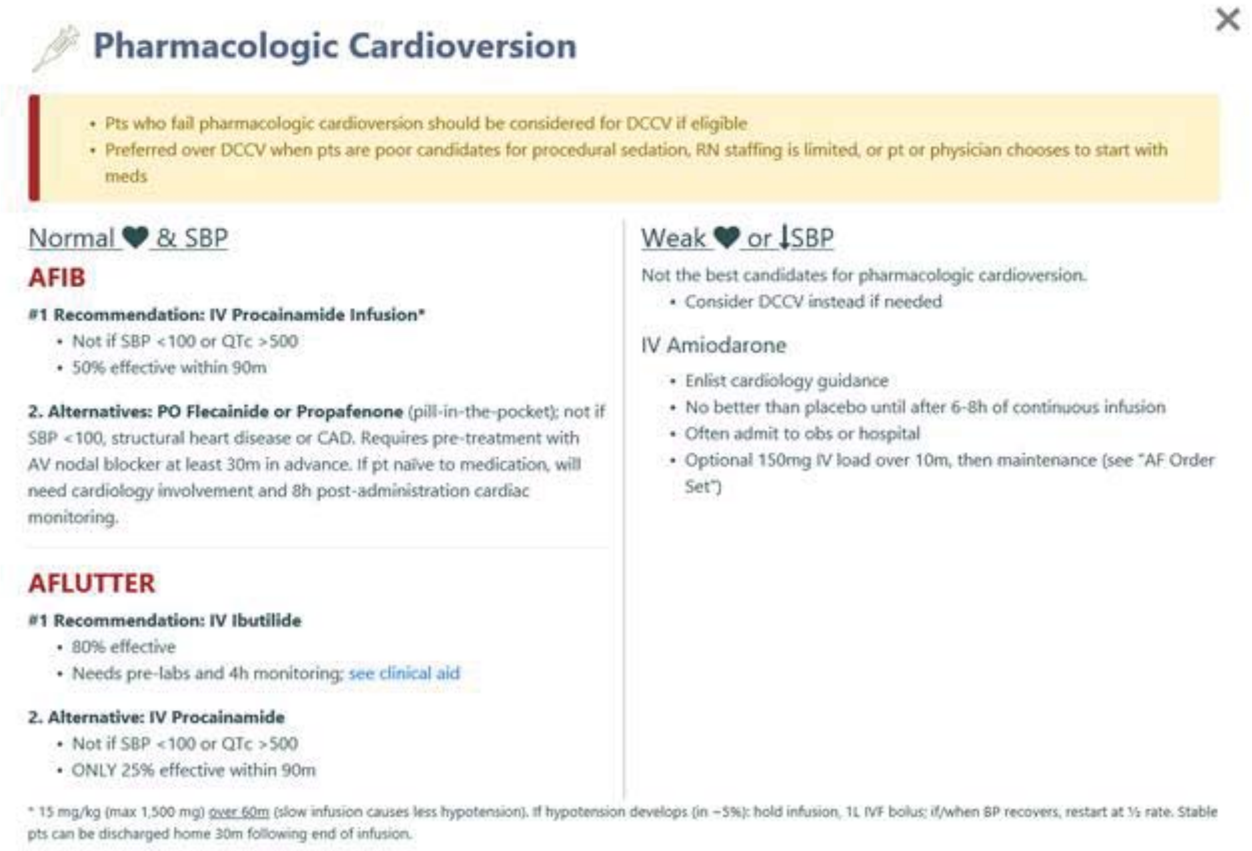


**Fig. 5**. Pharmacologic cardioversion screen in RISTRA-AF

A weak heart is defined as 1 with an ejection fraction less than 50% (based on recent echocardiography or physician gestalt) or clinical evidence of decompensated heart failure.

AFIB, atrial fibrillation; AFLUTTER, atrial flutter; AV, atrioventricular; BP, blood pressure; CAD, coronary artery disease; DCCV, direct current cardioversion; IV, intravenous; IVF, intravenous fluid; max, maximum; med, medication; obs, observation unit; pt, patient; pre-labs, pre-treatment laboratory testing; QTc, corrected QT interval; RN, registered nurse; SBP, systolic blood pressure.

Our medication recommendations are stratified by rhythm (AF vs atrial flutter), structural heart disease (good vs weak hearts, as defined above) and systolic blood pressure. For normotensive patients without known structural heart disease, we suggest IV procainamide for several reasons: it is easy to administer, has a good safety profile, has a relatively rapid effect (over 50% at 90 minutes), does not require prolonged monitoring (unlike IV ibutilide in all patients [4 hours], oral flecainide and oral propafenone in drug-naïve patients [8 hours]), and has been well studied among unselected ED patients with presumed recent-onset AF (<48 hours).[18, 69] Procainamide is the most common cardioversion medication used in Canadian EDs and the recommended drug-of-choice by the Canadian societies for eligible ED patients with recent-onset AF.[3, 20, 71]

Our second-line agents for pharmacologic cardioversion of hemodynamically stable ED patients with AF and good hearts are the oral agents propafenone and flecainide, famously used for the "pill-in-the-pocket" approach to rhythm control.[72-75] Though they may be more effective than

procainamide in restoring sinus rhythm, Class Ic agents require pre-treatment with atrioventricular nodal blockers and, on first use, cardiology involvement and at least 8 hours of cardiac monitoring, which in our system often involves admission to an observation or inpatient unit. What these medications gain in effectiveness, they lose in efficiency. If effective and safely tolerated in a monitored setting, these oral medications can subsequently be self-administered at home for the treatment of future paroxysmal AF episodes in select patients.[73]

For normotensive patients with atrial flutter and no known structural heart disease, ibutilide is our drug-of-choice because of its effectiveness over IV procainamide (approximately 62% vs 25% at 90 minutes).[70, 76] IV ibutilide administration requires careful patient selection and protocol adherence to reduce the risk of polymorphic ventricular tachycardia, which is rare if ibutilide is properly used.[70, 77] The median time to effect of IV procainamide and IV ibutilide (approximately 30-40 minutes) contrasts sharply with IV amiodarone, which fails to reliably outperform placebo for 6-8 hours.[19] This delay is not conducive to timely cardioversion and hampers departmental operational and resource efficiencies, often requiring admission to an observation unit or inpatient ward for administration. Because of its limitations, IV amiodarone for ED patients with AF or atrial flutter is reserved for those with hypotension, left ventricular ejection fraction ≤50% or decompensated heart failure, for whom IV procainamide and ibutilide, as well as oral flecainide and propafenone, are contraindicated. Because IV amiodarone recipients in our model of care are generally higher-risk patients, early cardiology consultation and inpatient monitoring are prudent to personalize safe management.

**Stroke Prevention**

One of the most serious complications of AF and atrial flutter is ischemic stroke, which can be significantly disabling, if not fatal. Fortunately, thromboprophylaxis can reduce stroke risk by two-thirds and mortality by 25%.[11, 54, 78] Stroke prevention is a critical component of AF and atrial flutter management in all society guidelines.[12, 79] The ED provides an important opportunity to identify patients who meet criteria for anticoagulation, and ED care may serve as a sentinel moment for behavioral change.[24, 80-82] Initiating stroke-prevention therapy at the time of ED discharge to home has been shown to be safe and associated with a mortality reduction.[83] Yet emergency physicians often under-prescribe anticoagulation on discharge of eligible patients with AF and atrial flutter.[80, 81, 84] In some health systems, patients interested in starting anticoagulation who receive a prescription at the time of ED discharge are more likely than their non-treated counterparts to be on anticoagulation 1 year later.[85] Several ED studies have used clinical decision support tools to increase ED prescribing of oral anticoagulants in eligible AF patients on discharge to home.[86, 87]

However, some have debated whether the initiation of anticoagulation at discharge for home-going patients falls within the scope of ED care.[88] What cannot be debated is the value of identifying at-risk patients with AF and atrial flutter and informing them that stroke prevention is an important topic worth exploring with their outpatient physicians. Even a brief discussion on

stroke prevention with an emergency physician may move eligible patients 1 step closer towards anticoagulation.

The $CHA_2DS_2$-VASc score is currently recommended in various society guidelines for stroke risk stratificiation.[11, 54] We opted to use it to identify patients at sufficient stroke risk to warrant anticoagulation, despite its significant shortcomings.[89, 90] To make the $CHA_2DS_2$-VASc score easier to use, we auto-populated it in RISTRA-AF by drawing in comorbidities from the EHR Problem List, as we have done with other clinical applications.[30, 91] All patients in RISTRA-AF receive a $CHA_2DS_2$-VASc calculation unless they have a stroke-prone condition in which anticoagulation is indicated regardless of their risk score: moderate-to-severe mitral stenosis, mechanical valve, or hypertrophic cardiomyopathy. In these higher-risk patients not currently on anticoagulation, we recommend a consult to the pharmacy-led telephone-based Anticoagulation Management Service.

If the patient has an elevated $CHA_2DS_2$-VASc score (≥2 in men and ≥3 in women), is not currently taking an anticoagulant, and will be discharged to home, we recommend they receive 1 or more of the following stroke prevention actions: (1) a risk-specific educational handout, reviewed at the bedside with the treating physician in a shared decision-making conversation. The handout is designed to be taken home as part of the patient's discharge instructions and can be used to facilitate discussion with family and with their outpatient physician (**Figure 6**); (2) if patients express interest in learning more about the benefits and risks of stroke prevention, the emergency physician can send an electronic consult to the Anticoagulation Management Service, which will contact eligible patients to discuss treatment options; (3) a 30-day prescription of an oral anticoagulant. Currently in our health system, dabigatran is the initially recommended anticoagulant for at-risk patients, if eligible. In RISTRA-AF, we provide guidance on dosing and contraindications and link the physician to a patient handout from the health system on the medication. If the physician wants to explore alternative anticoagulants, we provide links to internal resources on how to tailor the anticoagulant choice for patients with AF or atrial flutter.

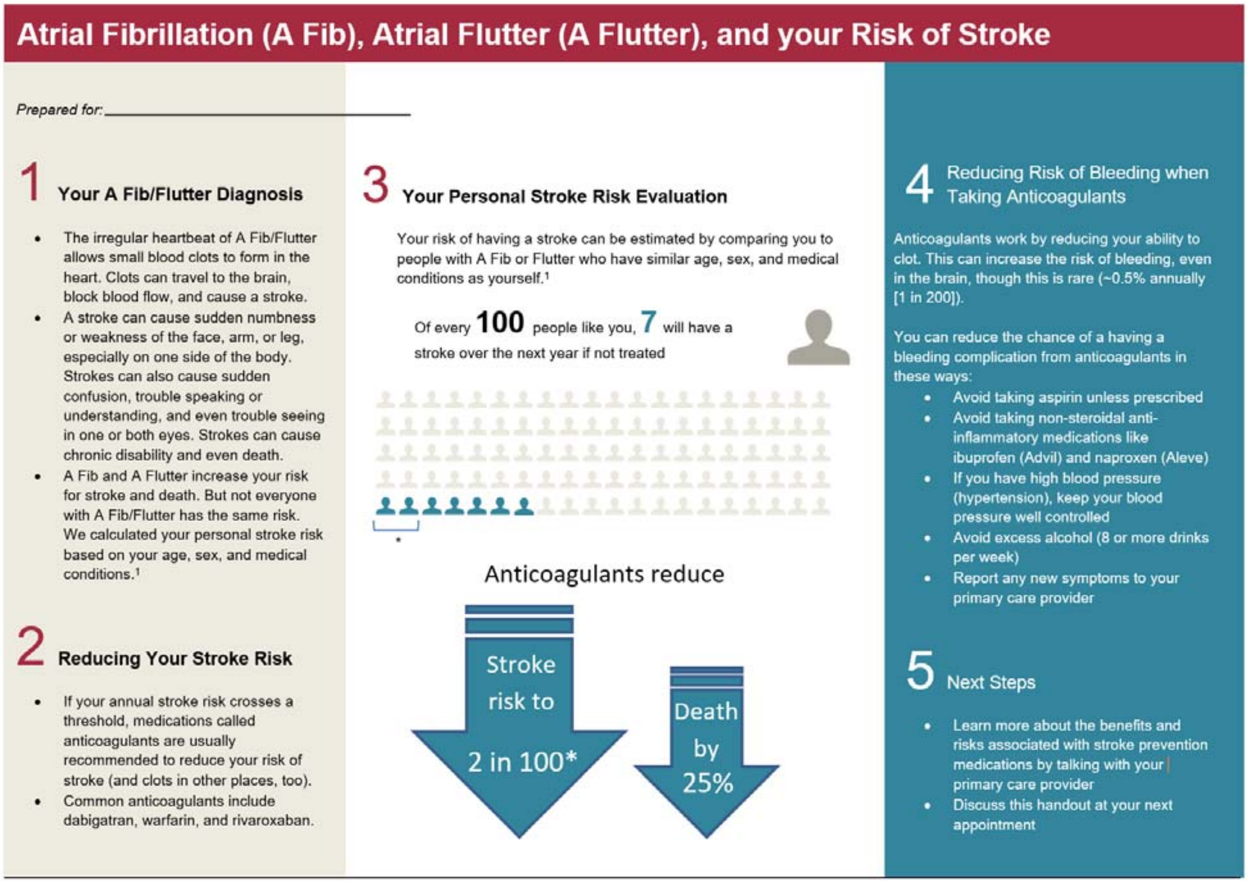


**Figure 6**. Patient-specific handout used in shared decision-making on stroke prevention with at-risk ED patients with AF or atrial flutter

Some emergency medicine pathways identify patients with AF or atrial flutter who are eligible for anticoagulation by using a high predicted stroke risk combined with a low estimated bleed risk, e.g., the HAS-BLED score.[92] We include on the anticoagulation screen a link to both the HAS-BLED score as well as a summary of how it was designed to be used. The fundamental purpose of HAS-BLED is to draw attention to reversible risk factors that need correcting rather than to exclude patients from being recommended anticoagulation if they are at increased risk for ischemic stroke; patients with a higher HAS-BLED score require more careful review and closer monitoring by their outpatient care team.[93]

### Follow-up after ED Discharge to Home

It is critical to patient care and outcomes that emergency physicians transfer care to outpatient physicians who can continue to manage rhythm-related symptoms via rate or rhythm control and to refer for cardiology management as needed, e.g., for complex cases or procedural intervention like elective outpatient cardioversion or ablation. An equally important component of ongoing primary care management is to proactively manage cardiovascular risk factors and comorbidities such as obesity, hypertension, and diabetes.[12, 20, 94, 95] We recommend that patients with AF or atrial flutter receive timely outpatient follow-up (<7 days) (**Figure 2**). Some multidisciplinary ED

treatment pathways for AF and atrial flutter create a new, dedicated outpatient clinic to facilitate post-ED follow-up.[87, 96, 97] Given our integrated health care delivery framework, health plan members have primary care physicians with whom timely follow-up is readily available (and those physicians have access to the same integrated EHR used in our EDs), so the creation of a specific AF clinic for discharged ED patients was unnecessary.

**Wrap-up and Summary**

RISTRA-AF provides physicians an efficient way to document a structured summary of their AF-related ED management using the wrap-up screen. This requires physician input about elements of ED care that we use to build a templated summary paragraph that can be copied from RISTRA-AF for pasting into the ED note of the EHR.

**Outcomes**

The primary outcome for aim 1 is hospitalization.[14, 15] This includes admission to the inpatient setting and to outpatient observation units. We selected this broad definition to distinguish hospitalization from discharge to home directly from the ED. We will undertake a sensitivity analysis using a stricter definition of hospitalization, which includes only admission to the inpatient setting.

Secondary outcomes for aim 1 include (a) discharge to home <24 hours of ED registration; (b) total length of stay in the ED and hospital; and (c) ED administration of a long-acting rate-reducing medication among patients who received any rate-reducing medication, oral or IV, short- or long-acting. Long-acting rate-reducing medications include oral diltiazem XR, metoprolol tartrate, metoprolol succinate, and atenolol and IV magnesium sulfate, 2g or more. We will undertake a sensitivity analysis in which only 4g or more of IV magnesium sulfate will count as a long-acting rate-reducing medication, as recommended in RISTRA-AF. We are not including amiodarone among our rate-reducing medications because amiodarone can also be used for cardioversion, and we cannot readily distinguish the 2 indications. Another secondary outcome for aim 1 is administration of continuous IV infusion of diltiazem or esmolol, which may be reduced in patients receiving early long-acting rate-reducing medications.

The primary outcome for aim 2 is anticoagulation initiation in eligible patients with AF or atrial flutter at the time of ED discharge to home or within the following 30 days. Eligibility includes an elevated $CHA_2DS_2$-VASc score (≥2 in men and ≥3 in women) in health plan members not currently taking anticoagulants who are being discharged to home directly from the ED. Current anticoagulation use is defined using EHR data. A patient is considered to be taking oral anticoagulation if (a) any oral anticoagulation prescription was filled in 45 days prior to the index encounter, (b) the supply of a filled prescription would include the index encounter date, or (c) active use of an oral anticoagulant was documented in the medication review during the index encounter or during the 30 days prior. A secondary outcome of aim 2 is electronic consultation of the Anticoagulant Management Service, independent of anticoagulation initiation in eligible

patients (defined above).

**Analysis**

Analysis of RISTRA-AF effectiveness will be based on comparison of intervention and control groups according to the stepped-wedge cluster randomized pragmatic trial design. All analyses for this stepped-wedge group randomized trial will be approached using mixed model regression methods. As this is a group-randomized trial and all groups will receive the intervention, all analyses will be done as intent-to-treat. Outcome and predictor measures were derived from the EHR, based on operational processes.

We will examine within- and between-cluster correlation over time to elucidate possible correlation structures, including possible time-decay in the correlation over time. While intraclass correlation and the number of repeated individuals in our cohort are expected to be low based on pilot study data, we will describe the intraclass correlation and churn rates over time and by cluster. We will use descriptive statistics to examine outcome trends over time overall, by cluster and by intervention status. Following the methods for open cohort stepped-wedge designs with binary outcomes outlined by Li et. al,[98] we will use mixed models to allow for clustering with appropriate correlation structures, adjusting for time effects, RISTRA-AF status, and possibly hospital-level fixed effects.

We estimate that our stepped-wedge design (with 9 clusters and 10 steps) will include approximately 3,240 adult ED encounters with primary AF or atrial flutter during the 10-month roll-out period. Based on pilot data, we expect at least 1,886 patients in the usual care condition and 1,534 patients in the intervention condition. Using preliminary data at the pilot sites and the trial sites, baseline initial hospitalization rate was 26.6%. We estimate a minimally detectable 3% absolute difference in initial hospitalization rate (Aim 1) at a level of 90% power and a 2-sided test at the 2.5% significance level.

We estimated the minimum number of clusters needed to achieve 90% power based on pilot data. We present our most conservative estimates here. For the hospitalization outcome, we assume an average of 38 eligible encounters per cluster, intraclass correlation of 0.01, the cluster autocorrelation of 0.47, and the individual autocorrelation of 0.9 with a discrete-time decay, a churn-rate of 0.942 and adjustment for 1 cluster-level variable (annual ED census) with $R^2$ of 0.07.

Given that only 18% of ED encounters are eligible for stroke prevention action (discharged to home, current KP member, not currently or recently taking oral anticoagulants, and at high risk for stroke), the overall numbers of eligible encounters for the stroke-prevention related outcomes are much smaller. For the primary Aim 2 outcome (any prescription ordered for oral anticoagulation medications within 30 days of the index visit), power is still adequate in this study design to identify changes in rates of prescriptions ordered as small as 5% in the eligible subgroup. Based on pilot data and assuming an average of 7 eligible encounters per cluster, intraclass correlation of 0.006, the cluster autocorrelation of 0.356, and the individual autocorrelation coefficient of 0 with

a discrete-time decay, a churn-rate of 0.984 with no adjustments for cluster-level variables, our 9-cluster design will allow us to identify a 4.9% change in rates of anticoagulant prescription with 80% power.

We anticipate wide variation in practice patterns across our EDs, as we have seen in the management of other conditions.[99] Some EDs might start the trial further from their optimal performance level than others. These EDs may have more potential for practice improvement than others and more to gain from the intervention. To account for this, we will also report facility-specific changes from pre- to post-intervention, anticipating a larger impact at facilities whose pre-intervention practices were in the lower tertile.

Given the many variables we are collecting during this trial, we also will be able to address other important clinical questions. For example: What is the association of short-acting oral rate-reduction medications (e.g., diltiazem 30 mg) with hospitalization? Is timing of administration of long-acting rate-reducing medications (e.g., early vs late in the ED course) associated with ED length of stay and hospitalization? How does hospitalization prevalence compare between those receiving different doses of IV magnesium sulfate? What are the prevalence and effects of administering both non-dihydropyridine calcium channel blockers and beta-blockers? Was the trial intervention associated with a change in cardioversion prevalence and success and in selection of cardioversion agents (e.g., electrical vs pharmacologic; procainamide vs ibutilide) for AF and atrial flutter? What is the association of stroke prevention actions in the ED with the short- and long-term incidence of ischemic stroke and death among patients eligible for anticoagulation on ED discharge to home? Was the trial intervention associated with other measures of patient care recommended by RISTRA-AF, e.g., ordering of thyroid stimulating hormone and echocardiography testing when indicated?

## References


1. Rozen G, Hosseini SM, Kaadan MI, et al. Emergency Department Visits for Atrial Fibrillation in the United States: Trends in Admission Rates and Economic Burden From 2007 to 2014. J Am Heart Assoc 2018;7.
2. Staerk L, Sherer JA, Ko D, Benjamin EJ, Helm RH. Atrial Fibrillation: Epidemiology, Pathophysiology, and Clinical Outcomes. Circ Res 2017;120:1501-1517.
3. Rogenstein C, Kelly AM, Mason S, et al. An international view of how recent-onset atrial fibrillation is treated in the emergency department. Acad Emerg Med 2012;19:1255-1260.
4. Tran C, Bennell MC, Qiu F, et al. Predictors and clinical outcomes of inpatient versus ambulatory management after an emergency department visit for atrial fibrillation: A population-based study. Am Heart J 2016;173:161-169.
5. Stiell IG, Clement CM, Brison RJ, et al. Variation in management of recent-onset atrial fibrillation and flutter among academic hospital emergency departments. Ann Emerg Med 2011;57:13-21.
6. Barrett TW, Self WH, Jenkins CA, et al. Predictors of regional variations in hospitalizations following emergency department visits for atrial fibrillation. Am J Cardiol 2013;112:1410-1416.
7. Funk AM, Kocher KE, Rohde JM, et al. Variation in practice patterns among specialties in the acute management of atrial fibrillation. BMC Cardiovasc Disord 2015;15:21.
8. McAlister FA, Rowe BH. Variations in the emergency department management of atrial fibrillation: Lessons to be learned. Am Heart J 2016;173:159-160.
9. Sutherland K, Levesque JF. Unwarranted clinical variation in health care: Definitions and proposal of an analytic framework. J Eval Clin Pract 2020;26:687-696.
10. Stiell IG, Macle L. Canadian Cardiovascular Society atrial fibrillation guidelines 2010: management of recent-onset atrial fibrillation and flutter in the emergency department. Can J Cardiol 2011;27:38-46.
11. January CT, Wann LS, Calkins H, et al. 2019 AHA/ACC/HRS Focused Update of the 2014 AHA/ACC/HRS Guideline for the Management of Patients With Atrial Fibrillation: A Report of the American College of Cardiology/American Heart Association Task Force on Clinical Practice Guidelines and the Heart Rhythm Society. J Am Coll Cardiol 2019;74:104-132.
12. Hindricks G, Potpara T, Dagres N, et al. 2020 ESC Guidelines for the diagnosis and management of atrial fibrillation developed in collaboration with the European Association for Cardio-Thoracic Surgery (EACTS): The Task Force for the diagnosis and management of atrial fibrillation of the European Society of Cardiology (ESC) Developed with the special contribution of the European Heart Rhythm Association (EHRA) of the ESC. Eur Heart J 2021;42:373-498.
13. Kea B, Warton EM, Ballard DW, et al. Predictors of Acute Atrial Fibrillation and Flutter Hospitalization across 7 U.S. Emergency Departments: A Prospective Study. J Atr Fibrillation 2021;13:2355.
14. DeMeester S, Hess RA, Hubbard B, LeClerc K, Ferraro J, Albright JJ. Implementation of a Novel Algorithm to Decrease Unnecessary Hospitalizations in Patients Presenting to a Community Emergency Department With Atrial Fibrillation. Acad Emerg Med 2018;25:641-649.
15. De Leon E, Duan L, Rippenberger E, Sharp AL. Impact of Standardizing Management of Atrial Fibrillation with Rapid Heart Rate in the Emergency Department. Perm J 2018;22:17-049.
16. Voskoboinik A, Moskovitch J, Plunkett G, et al. Cardioversion of atrial fibrillation in obese patients: Results from the Cardioversion-BMI randomized controlled trial. J Cardiovasc Electrophysiol 2019;30:155-161.
17. Vinson DR, Isaacs DJ, Othieno AA, Liu TI. Improving first shock success in patients with atrial fibrillation undergoing electrical cardioversion. Europace 2019;21:833.

**18.** Stiell IG, Sivilotti MLA, Taljaard M, et al. Electrical versus pharmacological cardioversion for emergency department patients with acute atrial fibrillation (RAFF2): a partial factorial randomised trial. Lancet 2020;395:339-349.

**19.** Chevalier P, Durand-Dubief A, Burri H, Cucherat M, Kirkorian G, Touboul P. Amiodarone versus placebo and class Ic drugs for cardioversion of recent-onset atrial fibrillation: a meta-analysis. J Am Coll Cardiol 2003;41:255-262.

**20.** Andrade JG, Aguilar M, Atzema C, et al. The 2020 Canadian Cardiovascular Society/Canadian Heart Rhythm Society Comprehensive Guidelines for the Management of Atrial Fibrillation. Can J Cardiol 2020;36:1847-1948.

**21.** Brieger D, Amerena J, Attia JR, et al. National Heart Foundation of Australia and Cardiac Society of Australia and New Zealand: Australian clinical guidelines for the diagnosis and management of atrial fibrillation 2018. Med J Aust 2018;209:356-362.

**22.** Schott SL, Berkowitz J, Dodge SE, et al. Personalized, Electronic Health Record-Integrated Decision Aid for Stroke Prevention in Atrial Fibrillation: A Small Cluster Randomized Trial and Qualitative Analysis of Efficacy and Acceptability. Circ Cardiovasc Qual Outcomes 2021;14:e007329.

**23.** Atzema CL, Jackevicius CA, Chong A, et al. Prescribing of oral anticoagulants in the emergency department and subsequent long-term use by older adults with atrial fibrillation. CMAJ 2019;191:E1345-E1354.

**24.** Vinson DR, Kea B, Coll-Vinent B, Barrett TW, Atzema CL. Enlisting Emergency Medicine Clinicians to Help Reduce Strokes in High-Risk Patients With Atrial Fibrillation and Flutter. Clin Pharmacol Ther 2018;104:613-614.

**25.** Damen NL, Van den Bemt BJF, Hersberger KE, et al. Creating an Interprofessional guideline to support patients receiving oral anticoagulation therapy: a Delphi exercise. Int J Clin Pharm 2019;41:1012-1020.

**26.** Simon J, Hawes E, Deyo Z, Bryant Shilliday B. Evaluation of prescribing and patient use of target-specific oral anticoagulants in the outpatient setting. J Clin Pharm Ther 2015;40:525-530.

**27.** Atzema CL, Yu B, Schull MJ, et al. Association of Follow-Up Care With Long-Term Death and Subsequent Hospitalization in Patients With Atrial Fibrillation Who Receive Emergency Care in the Province of Ontario. Circ Arrhythm Electrophysiol 2019;12:e006498.

**28.** Vinson DR. Redressing Underrecognition of "Cold Drink Heart": Patients Teaching Physicians about Atrial Fibrillation Triggered by Cold Drink and Food. Perm J 2020;24.

**29.** Vinson DR, Mark DG, Chettipally UK, et al. Increasing safe outpatient management of emergency department patients with pulmonary embolism: a controlled pragmatic trial. Ann Intern Med 2018;169:855-865.

**30.** Mark DG, Huang J, Kene MV, et al. Prospective Validation and Comparative Analysis of Coronary Risk Stratification Strategies Among Emergency Department Patients With Chest Pain. J Am Heart Assoc 2021;10:e020082.

**31.** Hemming K, Haines TP, Chilton PJ, Girling AJ, Lilford RJ. The stepped wedge cluster randomised trial: rationale, design, analysis, and reporting. BMJ 2015;350:h391.

**32.** Loudon K, Treweek S, Sullivan F, Donnan P, Thorpe KE, Zwarenstein M. The PRECIS-2 tool: designing trials that are fit for purpose. BMJ 2015;350:h2147.

**33.** Gordon N, Lin T. The Kaiser Permanente Northern California Adult Member Health Survey. Perm J 2016;20:34-42.

**34.** Lieu TA, Platt R. Applied Research and Development in Health Care - Time for a Frameshift. N Engl J Med 2017;376:710-713.

**35.** Bornstein S. An integrated EHR at Northern California Kaiser Permanente: Pitfalls, challenges, and benefits experienced in transitioning. Appl Clin Inform 2012;3:318-325.

**36.** An J, Niu F, Zheng C, et al. Warfarin Management and Outcomes in Patients with Nonvalvular Atrial Fibrillation Within an Integrated Health Care System. J Manag Care Spec Pharm 2017;23:700-712.

37. Sheehan B, Nigrovic LE, Dayan PS, et al. Informing the design of clinical decision support services for evaluation of children with minor blunt head trauma in the emergency department: a sociotechnical analysis. J Biomed Inform 2013;46:905-913.
38. Ekstrom HL, Kharbanda EO, Ballard DW, et al. Development of a Clinical Decision Support System for Pediatric Abdominal Pain in Emergency Department Settings Across Two Health Systems Within the HCSRN. EGEMS (Wash DC) 2019;7:15.
39. Kharbanda AB, Vazquez-Benitez G, Ballard DW, et al. Effect of Clinical Decision Support on Diagnostic Imaging for Pediatric Appendicitis: A Cluster Randomized Trial. JAMA Netw Open 2021;4:e2036344-e2036344.
40. Masterson Creber RM, Dayan PS, Kuppermann N, et al. Applying the RE-AIM Framework for the Evaluation of a Clinical Decision Support Tool for Pediatric Head Trauma: A Mixed-Methods Study. Appl Clin Inform 2018;9:693-703.
41. Lugovskaya N, Vinson DR. Paroxysmal Atrial Fibrillation and Brain Freeze: A Case of Recurrent Co-Incident Precipitation From a Frozen Beverage. Am J Case Rep 2016;17:23-26.
42. Voskoboinik A, Marcus GM. The Impact of Alcohol Intake on Atrial Fibrillation. Curr Cardiol Rep 2020;22:111.
43. Ettinger PO, Wu CF, De La Cruz C, Jr., Weisse AB, Ahmed SS, Regan TJ. Arrhythmias and the "Holiday Heart": alcohol-associated cardiac rhythm disorders. Am Heart J 1978;95:555-562.
44. Groh CA, Faulkner M, Getabecha S, et al. Patient-reported triggers of paroxysmal atrial fibrillation. Heart Rhythm 2019;16:996-1002.
45. Voskoboinik A, Kalman JM, De Silva A, et al. Alcohol Abstinence in Drinkers with Atrial Fibrillation. N Engl J Med 2020;382:20-28.
46. Voskoboinik A, Kalman JM, Kistler PM. Caffeine and Arrhythmias: Time to Grind the Data. JACC Clin Electrophysiol 2018;4:425-432.
47. Fromm C, Suau SJ, Cohen V, et al. Diltiazem vs. Metoprolol in the Management of Atrial Fibrillation or Flutter with Rapid Ventricular Rate in the Emergency Department. J Emerg Med 2015;49:175-182.
48. Demircan C, Cikriklar HI, Engindeniz Z, et al. Comparison of the effectiveness of intravenous diltiazem and metoprolol in the management of rapid ventricular rate in atrial fibrillation. Emerg Med J 2005;22:411-414.
49. Bouida W, Beltaief K, Msolli MA, et al. Low-dose Magnesium Sulfate Versus High Dose in the Early Management of Rapid Atrial Fibrillation: Randomized Controlled Double-blind Study (LOMAGHI Study). Acad Emerg Med 2019;26:183-191.
50. Davey MJ, Teubner D. A randomized controlled trial of magnesium sulfate, in addition to usual care, for rate control in atrial fibrillation. Ann Emerg Med 2005;45:347-353.
51. Zaouche K, Mhadhbi H, Boubaker R, Baccouche R, Khattech I, Majed K. Magnesium Sulfate: an adjunctive therapy in the first hour of management of rapid atrial fibrillation in the emergency department. Tunis Med 2021;99:225-231.
52. Bhatti H, Mohmand B, Ojha N, C PC, R LC. The Role of Magnesium in the Management of Atrial Fibrillation with Rapid Ventricular Rate. J Atr Fibrillation 2020;13:2389.
53. Blecher GE, Stiell IG, Rowe BH, et al. Use of rate control medication before cardioversion of recent-onset atrial fibrillation or flutter in the emergency department is associated with reduced success rates. CJEM 2012;14:169-177.
54. January CT, Wann LS, Alpert JS, et al. 2014 AHA/ACC/HRS guideline for the management of patients with atrial fibrillation: a report of the American College of Cardiology/American Heart Association Task Force on Practice Guidelines and the Heart Rhythm Society. J Am Coll Cardiol 2014;64:e1-76.
55. Ballard DW, Reed ME, Singh N, et al. Emergency Department Management of Atrial Fibrillation and Flutter and Patient Quality of Life at One Month Postvisit. Ann Emerg Med 2015;66:646-654 e642.

**56.** Atzema CL. Atrial Fibrillation: Would You Prefer a Pill or 150 Joules? Ann Emerg Med 2015;66:655-657.

**57.** Pluymaekers N, Dudink E, Luermans J, et al. Early or Delayed Cardioversion in Recent-Onset Atrial Fibrillation. N Engl J Med 2019;380:1499-1508.

**58.** Vinson DR, Atzema CL. Early or Delayed Cardioversion in Recent-Onset Atrial Fibrillation. N Engl J Med 2019;381:386-387.

**59.** Botto GL, Tortora G. Is delayed cardioversion the better approach in recent-onset atrial fibrillation? Yes. Intern Emerg Med 2020;15:1-4.

**60.** Capucci A, Compagnucci P. Is delayed cardioversion the better approach in recent-onset atrial fibrillation? No. Intern Emerg Med 2020;15:5-7.

**61.** Boriani G, Imberti JF, Valenti AC, Malavasi VL, Vitolo M. Managing atrial fibrillation: the need for an individualized approach even in the emergency department. Intern Emerg Med 2020;15:9-12.

**62.** Andrade JG, Mitchell LB. Periprocedural Anticoagulation for Cardioversion of Acute Onset Atrial Fibrillation and Flutter: Evidence Base for Current Guidelines. Can J Cardiol 2019;35:1301-1310.

**63.** Wong BM, Perry JJ, Cheng W, et al. Thromboembolic events following cardioversion of acute atrial fibrillation and flutter: a systematic review and meta-analysis. CJEM 2021;23:500-511.

**64.** Stiell IG, McMurtry MS, McRae A, et al. Safe Cardioversion for Patients With Acute-Onset Atrial Fibrillation and Flutter: Practical Concerns and Considerations. Can J Cardiol 2019;35:1296-1300.

**65.** Warden BA, MacKay J, Jafari M, Willman A, Stecker EC. Use of Direct Oral Anticoagulants Among Patients Undergoing Cardioversion: The Importance of Timing Before Cardioversion. J Am Heart Assoc 2018;7:e010854.

**66.** Ramirez FD, Sadek MM, Boileau I, et al. Evaluation of a novel cardioversion intervention for atrial fibrillation: the Ottawa AF cardioversion protocol. Europace 2019;21:708-715.

**67.** Ramirez FD, Fiset SL, Cleland MJ, et al. Effect of Applying Force to Self-Adhesive Electrodes on Transthoracic Impedance: Implications for Electrical Cardioversion. Pacing Clin Electrophysiol 2016;39:1141-1147.

**68.** Oral H, Souza JJ, Michaud GF, et al. Facilitating transthoracic cardioversion of atrial fibrillation with ibutilide pretreatment. N Engl J Med 1999;340:1849-1854.

**69.** Stiell IG, Clement CM, Perry JJ, et al. Association of the Ottawa Aggressive Protocol with rapid discharge of emergency department patients with recent-onset atrial fibrillation or flutter. CJEM 2010;12:181-191.

**70.** Vinson DR, Lugovskaya N, Warton EM, et al. Ibutilide Effectiveness and Safety in the Cardioversion of Atrial Fibrillation and Flutter in the Community Emergency Department. Ann Emerg Med 2018;71:96-108.e102.

**71.** Andrade JG, Verma A, Mitchell LB, et al. 2018 Focused Update of the Canadian Cardiovascular Society Guidelines for the Management of Atrial Fibrillation. Can J Cardiol 2018;34:1371-1392.

**72.** Alboni P, Botto GL, Baldi N, et al. Outpatient treatment of recent-onset atrial fibrillation with the "pill-in-the-pocket" approach. N Engl J Med 2004;351:2384-2391.

**73.** Reiffel JA, Capucci A. "Pill in the Pocket" Antiarrhythmic Drugs for Orally Administered Pharmacologic Cardioversion of Atrial Fibrillation. Am J Cardiol 2021;140:55-61.

**74.** Echt DS, Ruskin JN. Use of Flecainide for the Treatment of Atrial Fibrillation. Am J Cardiol 2020;125:1123-1133.

**75.** Sestito A, Molina E. Atrial fibrillation and the pharmacological treatment: the role of propafenone. Eur Rev Med Pharmacol Sci 2012;16:242-253.

**76.** Stiell IG, Sivilotti MLA, Taljaard M, et al. A randomized, controlled comparison of electrical versus pharmacological cardioversion for emergency department patients with acute atrial flutter. CJEM 2021;23:314-324.

**77.** Domanovits H, Schillinger M, Thoennissen J, et al. Termination of recent-onset atrial fibrillation/flutter in the emergency department: a sequential approach with intravenous ibutilide and external electrical cardioversion. Resuscitation 2000;45:181-187.

**78.** Hart RG, Pearce LA, Aguilar MI. Meta-analysis: antithrombotic therapy to prevent stroke in patients who have nonvalvular atrial fibrillation. Ann Intern Med 2007;146:857-867.

**79.** Mehta NK, Strickling J, Mark E, et al. Beyond cardioversion, ablation and pharmacotherapies: Risk factors, lifestyle change and behavioral counseling strategies in the prevention and treatment of atrial fibrillation. Prog Cardiovasc Dis 2021;66:2-9.

**80.** Vinson DR, Warton EM, Mark DG, et al. Thromboprophylaxis for patients with high-risk atrial fibrillation and flutter discharged from the emergency department. West J Emerg Med 2018;19:346-360.

**81.** Kea B, Waites BT, Lin A, et al. Practice Gap in Atrial Fibrillation Oral Anticoagulation Prescribing at Emergency Department Home Discharge. West J Emerg Med 2020;21:924-934.

**82.** Boudreaux ED, Bock B, O'Hea E. When an event sparks behavior change: an introduction to the sentinel event method of dynamic model building and its application to emergency medicine. Acad Emerg Med 2012;19:329-335.

**83.** Coll-Vinent B, Martin A, Sanchez J, et al. Benefits of Emergency Departments' Contribution to Stroke Prophylaxis in Atrial Fibrillation: The EMERG-AF Study (Emergency Department Stroke Prophylaxis and Guidelines Implementation in Atrial Fibrillation). Stroke 2017;48:1344-1352.

**84.** Kea B, Alligood T, Robinson C, Livingston J, Sun BC. Stroke Prophylaxis for Atrial Fibrillation? To Prescribe or Not to Prescribe-A Qualitative Study on the Decisionmaking Process of Emergency Department Providers. Ann Emerg Med 2019;74:759-771.

**85.** Atzema CL, Austin PC, Chong AS, Dorian P, Jackevicius CA. The Long-Term Use of Warfarin Among Atrial Fibrillation Patients Discharged From an Emergency Department With a Warfarin Prescription. Ann Emerg Med 2015;66:347-354 e342.

**86.** Parkash R, Magee K, McMullen M, et al. The Canadian Community Utilization of Stroke Prevention Study in Atrial Fibrillation in the Emergency Department (C-CUSP ED). Ann Emerg Med 2019;73:382-392.

**87.** Barbic D, DeWitt C, Harris D, et al. Implementation of an emergency department atrial fibrillation and flutter pathway improves rates of appropriate anticoagulation, reduces length of stay and thirty-day revisit rates for congestive heart failure. CJEM 2018;20:392-400.

**88.** Barrett TW, Marill KA. Anticoagulation for emergency department patients with atrial fibrillation: is our duty to inform or prescribe? Ann Emerg Med 2013;62:566-568.

**89.** Singer DE, Chang Y, Borowsky LH, et al. A new risk scheme to predict ischemic stroke and other thromboembolism in atrial fibrillation: the ATRIA study stroke risk score. J Am Heart Assoc 2013;2:e000250.

**90.** van den Ham HA, Klungel OH, Singer DE, Leufkens HG, van Staa TP. Comparative Performance of ATRIA, CHADS2, and CHA2DS2-VASc Risk Scores Predicting Stroke in Patients With Atrial Fibrillation: Results From a National Primary Care Database. J Am Coll Cardiol 2015;66:1851-1859.

**91.** Vinson DR, Morley JE, Huang J, et al. The Accuracy of an Electronic Pulmonary Embolism Severity Index Auto-Populated from the Electronic Health Record: Setting the stage for computerized clinical decision support. Appl Clin Inform 2015;6:318-333.

**92.** Schwab K, Smith R, Wager E, et al. Identification and early anticoagulation in patients with atrial fibrillation in the emergency department. Am J Emerg Med 2021;44:315-322.

**93.** Lip GY, Lane DA. Bleeding risk assessment in atrial fibrillation: observations on the use and misuse of bleeding risk scores. J Thromb Haemost 2016;14:1711-1714.

**94.** Proietti M, Romiti GF, Olshansky B, Lane DA, Lip GYH. Improved Outcomes by Integrated Care of Anticoagulated Patients with Atrial Fibrillation Using the Simple ABC (Atrial Fibrillation Better Care) Pathway. Am J Med 2018;131:1359-1366.e1356.

**95.** Yoon M, Yang PS, Jang E, et al. Improved Population-Based Clinical Outcomes of Patients with Atrial Fibrillation by Compliance with the Simple ABC (Atrial Fibrillation Better Care) Pathway for Integrated Care Management: A Nationwide Cohort Study. Thromb Haemost 2019;119:1695-1703.

**96.** Rezazadeh S, Chew DS, Miller RJH, et al. Effects of a reminder to initiate oral anticoagulation in patients with atrial fibrillation/atrial flutter discharged from the emergency department: REMINDER study. CJEM 2018;20:841-849.

**97.** Zimetbaum P, Reynolds MR, Ho KK, et al. Impact of a practice guideline for patients with atrial fibrillation on medical resource utilization and costs. Am J Cardiol 2003;92:677-681.

**98.** Li F, Hughes JP, Hemming K, Taljaard M, Melnick ER, Heagerty PJ. Mixed-effects models for the design and analysis of stepped wedge cluster randomized trials: An overview. Stat Methods Med Res 2021;30:612-639.

**99.** Vinson DR, Ballard DW, Huang J, et al. Outpatient Management of Emergency Department Patients With Acute Pulmonary Embolism: Variation, Patient Characteristics, and Outcomes. Ann Emerg Med 2018;72:62-72 e63.
