# Supplementary material for: Decision Support Intervention and Anticoagulation for Emergency Department Atrial Fibrillation: The O’CAFÉ Stepped-Wedge Cluster Randomized Clinical Trial
Source: JAMA Netw Open. 2024 Nov 6;7(11):e2443097. doi: 10.1001/jamanetworkopen.2024.43097 (PMC11541643; doi:10.1001/jamanetworkopen.2024.43097)
Supplement: Supplement 3. — Data Sharing Statement [file jamanetwopen-e2443097-s003.pdf]

# Data Sharing Statement

Vinson. Decision Support Intervention and Anticoagulation for Emergency Department Atrial Fibrillation. *JAMA Netw Open*. Published November 06, 2024.
doi:10.1001/jamanetworkopen.2024.43097

**Data**
**Additional Information:** ClinicalTrials.gov Identifier NCT05009225
https://clinicaltrials.gov/study/NCT05009225
**Data available:** No

**Additional Information**
**Explanation for why data not available:** We will share aggregate data, but we are not able to share individual patient data.
